# Identification Markers Responsible for Differentially Processed *Polygonatum cyrtonema* Hua by Ultra-Performance Liquid Chromatography with Quadruple-Time-of-Flight Mass Spectrometry

**DOI:** 10.3390/molecules29071559

**Published:** 2024-03-30

**Authors:** Ruihua Nie, Cuihong Wu, Xuan Zhang, Pei Deng

**Affiliations:** 1 QiHuang Chinese Medicine Academy, Jiangxi University of Chinese Medicine, Nanchang 330025, China; 20131071@jxutcm.edu.cn; 2 Hebei Institute for Drug and Medical Device Control, Shijiazhuang 050227, China; biowch@163.com; 3 School of Chemistry & Chemical Engineering, Nanchang University, Nanchang 330031, China; zhang97081519@163.com; 4 School of Resources & Environment, Nanchang University, Nanchang 330031, China

**Keywords:** *Polygonatum cyrtonema* Hua, Nine-Steam-Nine-Bask, UPLC-Q/TOF-MS, maker, discrimination of P-PCHs

## Abstract

The rhizome of *Polygonatum cyrtonema* Hua has been used as a traditional Chinese medicine for over 2000 years. The fresh Chinese herb possesses micro toxicity and is thus traditionally alternately steamed and basked nine times to alleviate the toxicity and enhance the pharmaceutical efficacy. Different processing cycles usually result in variable therapeutic effects in the processed *Polygonatum cyrtonema* Hua (P-PCH). However, it can be hard to tell these various P-PCHs apart at present. To identify the P-PCHs that had undergone repeated steaming one to nine times, the chemical constituents were profiled based on Ultra-Performance Liquid Chromatography with Quadruple-Time-of-Flight Mass Spectrometry, and the Principal Component Analysis and Cluster Analysis methods were adopted to discriminate different cycles of P-PCH. A total of 44 characteristic markers were identified, which allowed the P-PCHs to be discriminated exactly.

## 1. Introduction

*Polygonatum cyrtonema* Hua (*P. cyrtonema*) belongs to the family *Liliaceae*. There are 31 species in China, widely distributed in most areas except the southern tropical regions [1]. The dried roots of *P. cyrtonema,* also known as ‘Huang Jing’ in China, have been used as a traditional Chinese medicine for over two thousand years [2]. *P. cyrtonema* has high medicinal and nutritional value due to its diverse array of chemical constituents, such as flavonoids [3], triterpenoid saponins [4], alkaloids, lignin, amino acids, quinones, vitamins, and polysaccharides [5]. Polysaccharides [6] and oligosaccharides [7] are the main bioactive constituents according to the published researches. Numerous pharmacological studies have identified various biological activities of this plant, including antioxidant, cytotoxic, antibiotic, and hypoglycemic activities [8,9,10]. The raw product is highly irritating and needs to be steamed and dried before it can be used as medicine. The general processing method of the main material in this study involved alternating steaming and basking nine times, which is called the ‘Nine-Steam-Nine-Bask’ method. ‘Nine-Steam-Nine-Bask’-processed products, known as ‘Jiu Zhi Huang Jing’ in China, have been extensively used as tonic medicines. It is difficult to identify the processed product due to the complicated processing technology. However, identification is key to the quality control of medicinal material. Generally, qualitative indexes relating to appearance and texture usually act as the identification standard during the steaming and basking process, such as darkness, softness, and especially non-bitterness. Therefore, the processed *Polygonatum cyrtonema* Hua (P-PCH) could meet some quality demands, such as being black and soft. Notably, while the rhizomes of *P. cyrtonema* experienced five cycles of alternating stewing and solarization, they tasted almost non-bitterness, which was the same as those processed by steaming with nine times. Therefore, the excellent product prepared by the ‘Nine-Steam-Nine-Bask’ technology cannot be identified only by some phenotypic traits. On another side, the quality of *P. cyrtonema* is evaluated by the polysaccharide content in Chinese Pharmacopoeia (2020 edition). It is reported that the polysaccharide content decreased gradually following repeated steaming and basking. However, it is almost stable after the fourth repeating cycle [11]. Until now, it is still impossible to distinguish whether the P-PCHs are processed with nine cycles by their texture or polysaccharide content. However, it is truly crucial for quality control. Therefore, to exactly identify P-PCHs processed by different cycles of steaming and basking, research on analyzing the chemical profile dynamically and understanding the changes in the chemical composition during the repeating procedures is urgent.

Commonly used analytical methods for plant extract include nuclear magnetic resonance (NMR) [12], gas chromatography (GC) [13], and liquid chromatography (LC) [14]. LC is the most widely used method owing to its various detectors, while NMR is not suitable for trace amounts of gradients, and GC requires tedious derivatization [15]. Liquid chromatography-mass spectrometry (LC–MS) is a powerful analytical technique used for the separation, identification, and quantification of both unknown and known compounds, as well as to elucidate the structure and chemical properties of different molecules. It is very useful for analyzing small molecules and offers higher sensitivity and selectivity in the trace analysis of multicomponent-containing substances. Therefore, it has wide usage in chemical component analysis for complicated plant extracts. In the present study, an efficient and sensitive method has been developed by UPLC-QTOF-MS. Additionally, the chemical constituents of fresh and processed rhizomes of *P. cyrtonema* have been comprehensively profiled. Moreover, the metabolomics approach was utilized to analyze the variation of chemical constituents during the preparation process. We successfully discriminate the ten kinds of *P. cyrtonema,* including the raw material and the processed herbs, by establishing corresponding chemical markers for each of them. These findings offer a material foundation for the quality control of P-PCHs.

## 2. Results and Discussion

### 2.1. Selection of Constituents Differentially Presented in R-PCH and P-PCHs

**Figure 1:**
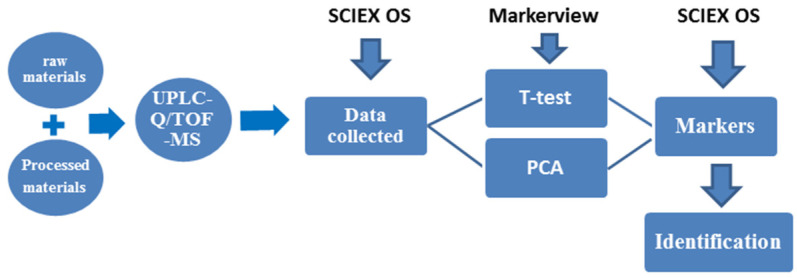
Scheme of the proposed strategy for screening markers.

**Figure 2:**
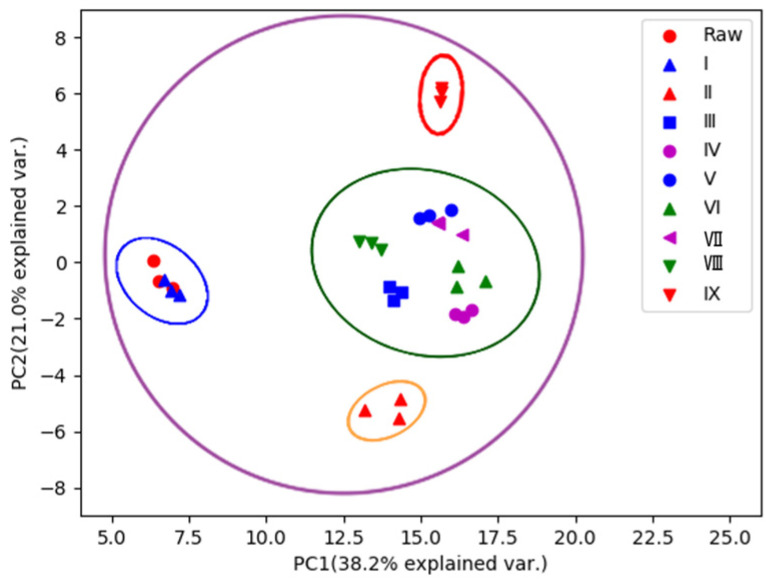
PCA scores plots of R-PCH and nine kinds of P-PCHs.

To identify the characteristic compounds specific to each R-PCH or P-PCH, the following strategy was developed (Figure 1). A total of 84 compounds were identified or tentatively characterized from raw *Polygonatum cyrtonema* Hua (R-PCH) and P-PCHs (Supplemental Material Table S1), and all of these compounds were probed among R-PCH and P-PCHs. The distribution pattern of the dereplicated metabolites among different extracts of the studied R-PCH and P-PCHs was then compared by PCA (Figure 2). This was generated from four components and explained 59.2% of the total variance, as the first principal component (PC1) and second principal component (PC2) accounted for 38.2% and 21% of the total variance, respectively. The differences among these were shown in the score scatter plot, which showed significant differences between R-PCH and P-PCHs. Furthermore, the score plot of PCA showed clear boundaries between R-PCH and other P-PCHs. It was evident that the ten groups of PCH samples were approximately displayed in four clusters related to the processing cycle (R-PCH and P-PCH I, P-PCH II, P-PCH III~VIII, and P-PCH IX). The cluster of P-PCH III~VIII was consistent with the results reported in the literature [16] that the quality of *P. cyrtonema* was not stable until processed by four cycles of steaming and basking. Nevertheless, the significant change in chemical constituents that happened in the P-PCH IX was almost ignored in previous research. Moreover, the isolated P-PCH IX demonstrated that the traditional ‘Nine-Steam-Nine-Bask’ processing technology was necessary for special active material foundation. In the above, radical changes happened to chemical constituents at points of the second steaming, third steaming, and ninth steaming, while the chemical components changed gradually from the fourth cycle to the eighth cycle. A total of 44 markers were obtained by the binary comparative analysis (Table 1). The compound that possessed *m*/*z* 373.1264 could serve as a marker for both R-PCH and P-PCH I, and could identify known compounds, especially those that were not reported in the *Polygonatum* genus. Taking the quantity of markers into consideration, P-PCH IX possessed the greatest quantity of characteristic compounds, which allowed it to be directly distinguished from other P-PCHs. Furthermore, it indicated that the traditional ‘Nine-Steam-Nine-Bask’ processing technology symbolized some special pharmaceutical constituents.

### 2.2. Chemical Structure Identification of Markers

The markers, such as *m*/*z* 1475.4740, 1313.4173, 1151.3651, and 989.3130, are tentatively characterized as oligosaccharides, which are selected for discriminating both R-PCH and P-PCH I from other P-PCHs. They are found in high levels in the raw medicinal material and P-PCH I; however, a decreasing tendency is observed starting from P-PCH II. A higher concentration of these compounds is observed in P-PCH I than in R-PCH, which indicates that the primary basking procedure possibly promotes the hydrolysis of polysaccharides. Notably, processing by steaming and basking alternatively might be crucial for the traditional technology.

Most of the flavonoids identified from *P. cyrtonema* are glycosides. The ESI-MS/MS has been widely used in the identification of flavonoids, and a vast number of experiments have been carried out to investigate the specific fragmentation patterns [17]. Therefore, a great deal of knowledge and experience was accumulated. The chemical structures are usually divided into three parts: A, B, and C rings [18]. It is proposed that the C ring easily experiences fragmentation processes I–IV (Supplemental Material Figure S1) at low collision energy when the B ring is substituted. While the B ring is not substituted, much more collision energy is required [19]. Additionally, methoxylated flavonoids tend to lose methyl radicals in ESI-MS/MS. According to these fragmentation pathways, the flavonoids explored in this study were tentatively characterized.

Compound 3, giving [M−H]^−^ ions at *m*/*z* 403.2483 in the negative MS spectrum, was characterized as (3α, 5β)-3-hydroxy-6,7-dioxocholan-24-oic acid (Supplemental material Figure S2). The product ion *m*/*z* 359.2576 was produced by the cleavage of the C-22 bond, and the base peak at *m*/*z* 343.2275 was attributed to further neutral loss of CH_4_ at the C-10 position from fragment at *m*/*z* 359.2576. The product ions *m*/*z* 367.2285 and *m*/*z* 311.2014 were formed by a neutral loss of H_2_O and cleavages of the OH-C_23_ bond and the C-20 bond, respectively. The fragment at *m*/*z* 113.0975 was observed tentatively by the cleavages of C-2 and C-4 bonds, and the charge was retained on the A-part [20]. Ultimately, it was tentatively identified as (3α, 5β)-3-hydroxy-6, 7-dioxocholan-24-oic acid.

**Figure 3:**
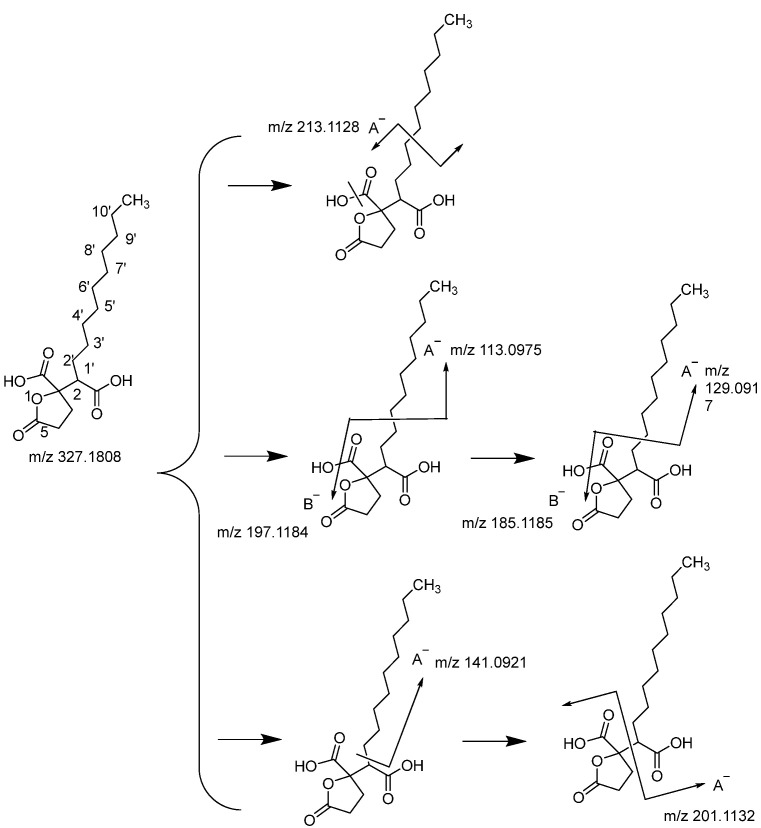
Fragmentation pathways of compound Spiculisporic acid.

The quasi-molecular ion at *m*/*z* 327.1808 supported the chemical formula C_17_H_28_O_6_ (Figure 3). The structure was deduced as Spiculisporic acid through fragmentation pattern analysis and combination with the ChemSpider database. Spiculisporic acid has been isolated from endophytic fungus *Aspergillus cejpii* [21,22]. An important fragment *m*/*z* 213.1128 was produced and the charge was retained on the A− by the neutral loss of H_2_O and the cleavage of the C-4′ bond. The most prominent fragmentation *m*/*z* 197.1184 occurred from a further cleavage of the C-3′ bond, while the charge was retained on the B−. The *m*/*z* 197.1184 neutral loss of C_8_H_16_ was also observed at *m*/*z* 113.0975, where its charge was retained on the A−. Similarly, the fragments at *m*/*z* 185.1185 and *m*/*z* 129.0917 were given by the further cleavage of the C-2′ bond, and charges were retained on the B- and A-, respectively. Fragments with *m*/*z* 144.0921 and *m*/*z* 201.1132 were formed through the cleavage of C-1′ and C-2.

**Figure 4:**
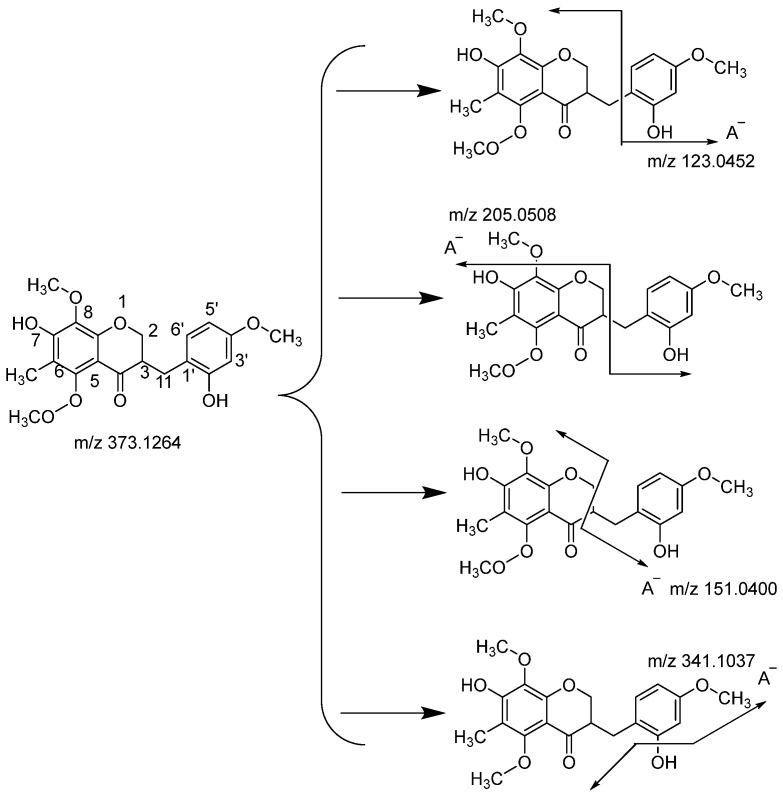
Fragmentation patterns of Ophiopogonanone F.

The compound possessing *m*/*z* 373.1264 was identified as Ophiopogonanone F [23] (Figure 4). According to the fragmental patterns of flavonoids, two methoxyls were probably located at the C-5 and C-8 positions, for which the A ring fragmentation was prohibited and no A ring fragment was observed. The product ion spectra giving a base peak at *m*/*z* 151.0400 might indicate that the cleavage of C-2 and C-3 bonds was the most prominent fragmentation pathway in which the charge was retained on the A−. Then, the fragment ion at *m*/*z* 205.0508 resulted from the cleavage of the C-3 bond and the loss of methoxyl in the C-8 position. Another product ion at *m*/*z* 123.0452 was produced by the cleavage of the C-11 bond, with the charge on the C ring. Uncommonly, the fragments at *m*/*z* 341.1037 were observed, owing to the cleavage of the C-7 and C-2 bonds, and the charge was retained on the A−.

The compound with *m*/*z* 315.0870 was discovered to be Homoferreirin [24,25] (Supplemental Material Figure S3) through diagnostic product ions (DPIs) screening analysis and fragmentation pathways analysis. As one of the most important fragmentation pathways in the ESI negative mode, the RDA C ring cleavage resulted in a substantial amount of product ions, and also acted as DPIs for flavonoid identification, such as C-1 and C-4 bonds cleavage, creating an A− product ion at *m*/*z* 125.0241. The product-ion spectra, with a base peak at *m*/*z* 151.0045, suggest that the C-1 and C-3 bond cleavages were the most prominent fragmentation. Then, the fragment ions at *m*/*z* 179.0349 corresponded to the cleavage of C-3 and C-1′, and a further neutral loss of H_2_O formed the *m*/*z* 163.0765, while the charges are retained on A− and B−, respectively. The product ions at *m*/*z* 81.0347 were formed by the cleavage of C-1′ and C-5′ bonds, while the charge is retained on A−. Lastly, the cleavage of C-3′ and C-5′ produced the fragment ion *m*/*z* 57.0346, and the charge was retained on A−.

## 3. Materials and Methods

### 3.1. Chemicals and Reagents

Acetonitrile of HPLC grade and formic acid with purity ≥ 99.7) was obtained from Merck (Darmstadt, Germany). Deionized water was obtained from the Millipore Milli-Q water system (Bedford, MA, USA). Other reagents were analytical grade.

### 3.2. Preparation of P-PCHs Samples

Fresh rhizomes of *P. cyrtonema* were collected from Poyang County, Jiangxi Province, China, and authenticated by Professor Yihao Jiang from Nanchang University. The voucher specimens were kept in our laboratory for future reference. The dried fresh rhizomes (2000 g) were autoclaved at a temperature of 100 °C for 4 h using a bamboo steamer, then cooled down, and then basked at 50 °C to a constant weight. One-tenth of the dried samples were collected for analysis and the remaining P-PCH was passed to the next steam treatment cycle.

### 3.3. Method for Extraction of P-PCHs Preparation

The dried rhizomes of P-PCHs were crushed into powder with a pulverizer (Heibei Benchen Technology Co., Ltd., Shijiazhuang, China). The dried powder (1 g) of each sample of P-PCHs was extracted with 80% methanol (10 mL) at 50 °C for 1 h in the ultrasonic auxiliary (Kunshan Ultrasound Instrument Co., Ltd., Kunshan, China). The methanol is analytical grade and purchased from Xilong Chemical Industry Incorporated Co., Ltd. (Guangzhou, China), and then centrifuged (12,000× *g* for 10 min; YingTai Instrument Co., Ltd., Changsha, China) to separate the supernatant from the residues. The top layer (1 mL) was collected for analysis.

### 3.4. Method for UPLC-QTOF-MS Analysis

Chromatographic separation was performed on a SCIEX X500R with BEH C18 column (2.1 × 100 mm, 1.7 μm), and the column was purchased from the Waters Corporation. The mobile phase consisted of 0.1% formic acid in water (A) and acetonitrile (B) with the following gradients: 0–2 min, 10% B; 2–2.5 min, 10–15% B; 2.5–3 min, 15–20% B; 3–3.5 min, 20% B; 3.5–4 min, 20–25% B; 4–4.5 min, 25–30% B; 4.5–5.0 min, 30–60% B; 5–9 min, 60–95% B; 9–11 min, 95% B; 11–11.5 min, 95–10% B; and 11.5–12.0 min, 10% B. The temperature of the column oven was set at 40 °C. The equilibration time and flow rate were set at 5 min and 0.3 mL/min, respectively. Each sample was injected three times in parallel with an injection volume of 0.2 μL.

The TOF MS operating conditions were as follows: ion source gas 1, 45 PSI; ion source gas 2, 50 PSI; temperature, 500 °C; spray voltage, −4500 V; TOF start mass, 100 Da; TOF stop mass, 1500 Da; delustering potential, −65 V; collision energy, −10 V; accumulation time, 0.1 s; intensity threshold exceeds, 10 cps. The MS/MS operating conditions were as follows: TOF start mass, 100 Da; TOF stop mass, 1500 Da; declustering potential, −80 V; collision energy, −40 V; CE spread, 15 V; and accumulation time, 0.065 s.

### 3.5. Data-Processing Method

TOF-IDA-MS/MS data was collected using SCIEX OS Version 1.6 software (SCIEX, Los Angeles, CA, USA), which could collect data, analyze results, and generate reports. To reduce chemical interference from the matrix, a mass tolerance for alignment of 10 ppm, retention tolerance of 0.50 min, maximum number of peaks of 5000, total intensity threshold of 100, and no isotope filtering were the filters set. Total peak area normalization was then applied to the retained peaks for the subsequent analysis. In order to find the significant differences between the P-PCHs, the Principal Component Analysis (PCA) and Cluster analysis models were built using MarkerView vision 1.3 (SCIEX, Los Angeles, CA, USA) software. Three repeated spectra for each sample were imported and analyzed with no scaling, and the number of predictors to try for each node was set to the square root of the total number of variables.

The results of the t-test indicate how well each variable distinguishes between the two groups. It was reported as a *p*-value (*p* < 0.05) [26] by the present study and realized by MarkerView. Chemical constituents featuring the largest fold-changes and smallest p-values are considered as significant differences between the two groups. To discriminate between the different P-PCHs, a pair-wise comparison of all the classes was performed by MarkerView Software (1.3 version) [27]. To obtain significantly altering metabolites related to the processing method, compounds with the features (*p* < 0.05, |log fold-change| > 1.5) of *t*-test were selected for analysis [28]. Take the P-PCH IX analysis as an example (Table 1). Firstly, the *t*-test comparative analysis was performed between R-PCH and P-PCH IX, P-PCH I and P-PCH IX, P-PCH II and P-PCH IX, P-PCH III and P-PCH IX, P-PCH IV and P-PCH IX, P-PCH V and P-PCH IX, P-PCH VI and P-PCH IX, P-PCH VII and P-PCH IX, and P-PCH VIII and P-PCH IX, respectively. In other words, to uncover the dynamic changes of chemical constituents during the ‘Nine-Steam-Nine-Bask’ process, pair-wise comparison of products from raw medicinal material to the last one was carried out. The differences in the chemical components between the raw medicinal material (R-PCH) and One-Steam-One-Bask product (P-PCH I) suggest variation occurs during the first preparation cycle. Then, the different chemical components between the One-Steam-One-Bask product (P-PCH I) and Two-Steam-Two-Bask product (P-PCH II) suggest the variation occurs during the second preparation cycle, and so on. The compounds with the features (*p* < 0.05, |log fold-change| > 1.5) were picked out from each group, of which the common constituents worked as markers unique to P-PCH IX. Likewise, markers unique to other P-PCHs were explored.

### 3.6. Structure Identification Method

The observed ions for all compounds and their respective calculated ions were used for both targeted identification of the known compounds and untargeted identification of some novel compounds. Strategies (Figure 1) based on the high-resolution MS/MS data and mass charge ratio, as well as retention rules (RTs), were adopted to identify the metabolites. Furthermore, the comparison was conducted using the TOF MS and databases such as ChemSpider (http://www.chemspider.com/, accessed on 6 January 2019), PubMed (https://www.ncbi.nlm.nih.gov/pubmed accessed on 6 January 2019), and MassBank (http://www.massbank.jp/Search, accessed on 6 January 2019). These could identify known compounds, especially those that were not reported in the *Polygonatum* genus.

## 4. Conclusions

The excellent traditional Chinese medicine of *P. cyrtonema* is processed by traditional technology of nine cycles of steaming and basking. However, this procedure is time-consuming and labor-intensive, and most drugs are made with fewer than nine cycles. Worse, there are no valid methods with which to identify them. To deal with the problem, a total of 44 characteristic markers have been founded which could be utilized to distinguish the different P-PCHs. In addition, it is worth mentioning that the dynamic change of chemical compositions is firstly examined, and significant differences between the different P-PCHs have been detected by principal component analysis (PCA). The chemical constituents were almost identical between the crude drug and P-PCHs with one cycle of steaming and basking. Abruptly, a great change occurred during the second steaming and basking, which allowed the characteristics of P-PCH II to be easily recognized. The third cycle product also exhibits obvious differentiation compared with the above samples. Interestingly, from the third cycle to the eighth cycle, the chemical constituents changed slightly. Furthermore, chemical constituents of the P-PCH IX are characteristic for dramatic change compared with those of the eighth cycle. Lastly, each kind of P-PCH has been identifiedl by the corresponding markers. This study firstly profiles the chemical constituents of processed *P. cyrtonema* and suggests a validated method for P-PCH identification, which seriously affects the quality control of *P. cyrtonema*. These findings also uncover the dynamic change of material foundation during the ‘Nine-Steam-Nine-Bask’ process for the first time.

## Figures and Tables

**Table 1:** Markers identified from raw and processed *P. cyrtonema*.

Group ^a^	No.	Rt (min)	*m*/*z* (Neg)	Formula	MS/MS (*m*/*z*)	Identification
0	1	2.10	177.0556	C_10_H_10_O_3_	65.0407, 93.0329, 134.0377, 161.0244, 162.0325, 175.0399, 176.0470, 177.0559	
0	2	7.66	265.1805	C_16_H_26_O_3_	55.0197, 80.0270, 96.9604, 124.0162, 233.1546, 265.1815	-
0	3	7.39	403.2483	C_24_H_36_O_5_	301.2170, 311.2014, 333.2448, 343.2275, 361.2401	(3alpha, 5beta)-3-hydroxy-6,7-dioxocholan-24-oic acid
0	4	8.12	424.3200	-	158.0603, 406.3111	-
0	5	7.16	476.2748	-	78.9589, 196.0381, 279.2318, 476.2783	-
0	6	7.94	466.2915	-	78.9592, 196.0375, 269.2481, 466.2953	-
0	7	6.77	800.4163	-	279.2339, 433.2353, 476.2789, 518.2911, 782.3837, 800.3846	-
0	8	6.78	799.4106	C_47_H_60_O_11_	179.0546, 591.3492, 753.4002	-
1	9	6.40	315.0870	C_17_H_16_O_6_	57.0340, 81.0347, 125.0241, 179.0349, 205.0877, 213.0566, 271.0998, 315.0869	-
1	10	6.81	373.1264	C_20_H_22_O_7_	123.0452, 151.0400, 205.0508, 341.1037	Ophiopogonanone F
1	11	6.12	945.4258	C_46_H_66_N_4_O_17_	179.0546, 737.4118, 899.4621	-
1	12	6.08	946.4296	-	738.4173, 900.4647	-
2	13	6.59	431.3374	C_24_H_48_O_6_	152.9952, 269.2044, 277.2173, 293.2106, 311.2042, 431.2443	Apigenin-7-*O*-glucoside
2	14	6.88	571.2886	C_28_H_40_N_6_O_7_	152.9960, 241.0119, 255.2332, 315.0493, 571.2900	-
3	15	1.43	622.2197	C_30_H_33_N_5_O_10_	118.0498, 160.0596, 190.0708, 262.0913, 280.1007	-
3	16	6.99	883.4660	-	89.0245, 179.0562, 721.4167, 883.4680	-
4	17	7.85	621.3621	C_30_H_50_N_4_O_6_	291.1972, 311.2231, 327.2203, 621.4388	-
4	18	7.30	767.4186	C_36_H_60_N_6_O_12_	119.0347, 247.0824, 575.3596, 721.4126	-
4	19	5.88	813.4248	-	89.0242, 179.0565, 767.4221, 813.4271	-
4	20	6.99	883.4660	-	89.0245, 179.0562, 721.4167, 883.4680	-
4	21	7.06	913.4755	C_47_H_74_O_18_	721.4193, 867.4757, 913.4847	-
4	22	6.99	929.4727	C_42_H_70_N_6_O_17_	89.0244, 179.0555, 721.4149, 737.4128, 883.4645	-
4	23	6.12	945.4258	C_46_H_66_N_4_O_17_	179.0546, 737.4118, 899.4621	-
4	24	5.81	959.4826	-	751.3942, 913.4454	-
5	25	7.03	327.1439	C_19_H_20_O_5_	149.0257, 177.0202, 178.0643, 205.0494, 206.0582, 327.1247	-
5	26	7.46	329.1597	C_16_H_26_O_7_	69.0345, 179.1803, 183.1769, 197.1908, 239.1654, 267.1627, 293.2121, 329.1679	Aurantio-obtusin
5	27	7.46	330.1639	-	57.0346, 180.1842, 198.1951, 214.1889, 268.1640, 325.1790	-
5	28	7.62	343.1761	C_17_H_28_O_7_	116.9275, 139.0060, 179.1815, 239.1654, 267.1557, 297.2436	3-hydroxy-13-tetradecene-1, 3, 4-tricarboxylic acid
5	29	7.3	767.4186	C_36_H_60_N_6_O_12_	119.0347, 247.0824, 575.3596, 721.4126	-
5	30	7.96	767.4222	C_51_H_60_O_6_	279.2329, 325.1839, 767.5078	-
6	31	8.43	425.2903	-	254.2514, 255.2404, 425.2594	-
6	32	6.99	883.4660	-	89.0245, 179.0562, 721.4167, 883.4680	-
7	33	7.47	267.1603	C_15_H_24_O_4_	82.0034, 125.9963, 179.1796, 195.1740, 239.1747, 267.1616	-
7	34	7.46	330.1639	-	57.0346, 180.1842, 198.1951, 214.1889, 268.1640, 325.1790	-
7	35	7.30	767.4186	-	89.0242, 205.0722, 721.4128	-
8	36	6.48	231.0098	C_15_H_4_O_3_	98.9656, 149.9747, 185.9510, 231.0127	-
8	37	6.01	365.0682	-	119.0515, 150.0300, 165.0557, 245.0114, 365.0686	-
8	38	7.77	399.2517	C_25_H_36_O_4_	161.0968, 193.1240, 327.2112, 399.2537	-
8	39	1.43	622.2197	C_30_H_33_N_5_O_10_	118.0498, 160.0596, 190.0708, 262.0913, 280.1007	-
9	40	6.64	327.1808	C_18_H_32_O_5_	129.0920, 141.0925, 171.1022, 185.1187, 197.1184, 201.1131, 213.1135, 291.1967, 327.2189	-
9	41	7.46	329.1597	C_16_H_26_O_7_	69.0345, 179.1803, 183.1769, 197.1908, 239.1654, 267.1627, 293.2121, 329.1679	Aurantioobtusin
9	42	7.46	330.1639	-	57.0346, 180.1842, 198.1951, 214.1889, 268.1640, 325.1790	-
9	43	6.77	327.1808	C_17_H_28_O6	87.0084, 197.1906, 225.1853, 251.1645	Spiculisporic acid
9	44	6.59	431.3374	C_21_H_20_O_10_	152.9952, 243.1596, 269.2044, 277.2173, 293.2106, 311.2042, 413.3272, 431.2443	Apigenin-7-*O*-glucoside

^a^ Markers in group 0, obtained from binary comparison between R-PCH and different P-PCHs, can be used to discriminate raw *P. cyrtonema*; likely markers in group 1 obtained from binary comparison between P-PCH I and R-PCH, and different P-PCHs, can be used to discriminate raw P-PCH I; markers classification in other groups (2–9) correspond to P-PCH II to P-PCH IX.

## Data Availability

Data are contained within the article.

## Supplementary Materials

The following supporting information can be downloaded at: https://www.mdpi.com/article/10.3390/molecules29071559/s1, Table S1: Chemical constituents identified by UPLC-QTOF-MS form R-PCH and P-PCHs. Table S2: The represent mark for each sample of R-PCH and P-PCHs. Figure S1: The proposed fragmental patterns of C ring for flavonoids. Figure S2: The plausible fragmentation pathways of compound 3. Figure S3: The plausible fragmentation pathways of Homoferreirin. Figure S4: The chemical markers for discrimination of R-PCH and other P-PCHs. Figure S5: The chemical markers for discrimination of PCH-I and other P-PCHs (including R-PCH). Figure S6: The chemical markers for discrimination of PCH-II and other P-PCHs (including R-PCH). Figure S7: The chemical markers for discrimination of PCH-III and other P-PCHs (including R-PCH). Figure S8: The chemical markers for discrimination of PCH-IV and other P-PCHs (including R-PCH). Figure S9: The chemical markers for discrimination of PCH-V and other P-PCHs (including R-PCH). Figure S10: The chemical markers for discrimination of PCH-VI and other P-PCHs (including R-PCH). Figure S11: The chemical markers for discrimination of PCH-VII and other P-PCHs (including R-PCH). Figure S12: The chemical markers for discrimination of PCH-VIII and other P-PCHs (including R-PCH). Figure S13: The chemical markers for discrimination of PCH-IX and other P-PCHs (including R-PCH). Figure S14: The extract ion chromatogram (XIC) of the marker 1475.4740 in thereof different P-PCHs and R-PCH, to clarify the dynamic changes in the process of steaming and basking. Figure S15: The extract ion chromatogram (XIC) of the marker 1313.4170 in thereof different P-PCHs and R-PCH, to clarify the dynamic changes in the process of steaming and basking. Figure S16: The extract ion chromatogram (XIC) of the marker 1151.3561 in thereof different P-PCHs and R-PCH, to clarify the dynamic changes in the process of steaming and basking. Figure S17: The extract ion chromatogram (XIC) of the marker 989.3130 in thereof different P-PCHs and R-PCH, to clarify the dynamic changes in the process of steaming and basking. Figure S18: The extract ion chromatogram (XIC) of the marker 403.2483 in thereof different P-PCHs and R-PCH, to clarify the dynamic changes in the process of steaming and basking. Figure S19: The extract ion chromatogram (XIC) of the marker 327.1808 in thereof different P-PCHs and R-PCH, to clarify the dynamic changes in the process of steaming and basking. Figure S20: The extract ion chromatogram (XIC) of the marker 373.1264 in thereof different P-PCHs and R-PCH, to clarify the dynamic changes in the process of steaming and basking. Figure S21: The extract ion chromatogram (XIC) of the marker 315.0870 in thereof different P-PCHs and R-PCH, to clarify the dynamic changes in the process of steaming and baskin.
