# Supplementary material for: Identification Markers Responsible for Differentially Processed Polygonatum cyrtonema Hua by Ultra-Performance Liquid Chromatography with Quadruple-Time-of-Flight Mass Spectrometry
Source: Molecules. 2024 Mar 30;29(7):1559. doi: 10.3390/molecules29071559 (PMC11013794; doi:10.3390/molecules29071559)
Supplement: Supplementary file 1 [file molecules-29-01559-s001.zip › molecules-2898733-supplementary.pdf]

Supplemental material

Identification Markers Responsible for Differentially Processed *Polygonatum cyrtonema* Hua by Ultra-Performance Liquid Chromatography with Quadruple-Time-of-Flight Mass Spectrometry

Ruihua Nie [1], Cuihong Wu [2], Xuan Zhang [3] and Pei Deng [4,*]

[1] QiHuang Chinese Medicine Academy, Jiangxi University of Chinese Medicine, Nanchang 330025, China; 20131071@jxutcm.edu.cn
[2] Hebei Institute for Drug and Medical Device Control, Shijiazhuang 050227, China; biowch@163.com
[3] School of Chemistry & Chemical Engineering, Nanchang University, Nanchang 330031, China; zhang97081519@163.com
[4] School of Resources & Environment, Nanchang University, Nanchang 330031, China
* Correspondence: dengpei@ncu.edu.cn

Table S1 chemical constituents identified by UPLC-QTOF-MS form R-PCH and P-PCHs.

**No.**	**Rt (min)**	***m/z* (Neg)**	**Formula**	**MS/MS (*m/z*)**	**Identification**
1	2.15	177.0556	$C_{10}H_{10}O_3$	65.0407, 93.0329, 134.0377, 161.0244, 162.0325, 175.0399, 176.0470, 177.0559	-
2	7.66	265.1805	$C_{16}H_{26}O_3$	55.0197, 80.0270, 96.9604, 124.0162, 233.1546, 265.1815	Isomer of tetranor-12(*R*)-HETE
1	0.76	173.1041	$C_6H_{14}N_4O_2$	58.0411,83.0614,131.0827,131.1007	L-(+)-Arginine
2	0.78	827.2678	$C_{30}H_{52}O_{26}$	179.0558,341.1084,545.1719,827.2644	Isomer of Maltopentose
3	1.52	133.0139	$C_4H_6O_5$	59.0134,71.0137,72.9928,115.0031	Malic acid
4	3.27	210.0762	$C_{10}H_{13}NO_4$	94.0296,106.0296,124.0402	Isomer of methyldopa
5	5.54	637.2135	$C_{30}H_{38}O_{15}$	179.0346,207.0667,313.1081,637.2152	5,3′-Dimethyl hesperidin
6	5.63	523.2384	$C_{23}H_{40}O_{13}$	113.0240,161.0457,315.1809,477.2336	-
7	5.69	282.1118	$C_{17}H_{17}NO_3$	119.0496,162.0555,174.0554,282.1119	Trans-N-*p*-coumaroyl tyramine-
8	5.74	243,1240	$C_{14}H_{12}O_4$	125.0984,199.1353,207.1025,225.1139	Oxyresveratrol
9	5.78	312.1222	$C_{18}H_{19}NO_4$	135.0451,148.0531,190.0511,297.1004	-
10	6.01	365.0682	-	119.0499,150.0317,165.0552,245.0117	-
11	6.08	301.0706	$C_{16}H_{14}O_6$	121.0295,125.0240,179.0345,191.0345	Isomer of hesperetin
12	6.13	285.0399	$C_{15}H_{10}O_6$	133.0296,149.0238,199.0401,285.0397	Kaempferol
13	6.19	393.1946	$C_{19}H_{22}O_9$	79.9571,191.0741,313.1077,393.1938	Hydroxymusizin-hexose
14	6.26	331.2468	-	139.1120,171.1035,211.1337,331.2399	-
15	6.26	329.2322	-	99.0810,139.1123,171.1017,211.1324	-
16	6.40	299.0914	$C_{17}H_{16}O_5$	95.0133,149.0601,165.0183,193.0501	Isomer of 7-hydroxy-5,8-dimethoxy-2-phenyl-2,3-dihydro-4h-chromen-4-one
17	6.40	375.1844	-		-

				80.9655,170.9400,293.2134,375.1843	
18	6.49	301.1075	$C_{17}H_{18}O_5$	153.0558,166.0272,180.0425,195.0655	Isomer of mucronulatol
19	6.49	329.0997	$C_{18}H_{18}O_6$	71.0498,97.0293,139.0393，193.0505	Isomer of 3-hydroxy-5,7-dimethoxy-2-(4-methoxyphenyl)-2,3-dihydro-4h-chromen-4-one
20	6.49	301.1075	$C_{15}H_{10}O_7$	93.0347,166.0272,180.0425,195.0655	Delphinidin
21	6.66	341.2326	$C_{12}H_{22}O_{11}$	59.0129,154.1018,187.0946,291.1940	Sucrose
22	6.83	311.2212	$C_{18}H_{32}O_4$	57.0340，113.0961，171.1030，183.0126，197.1184，211.1343,293.2134，311.2229	Isomer of succinic acid
23	6.65	343.2119	$C_{18}H_{32}O_6$		-
24	6.88	385.2585	$C_{21}H_{38}O_6$	249.2223，293.2118，311.2224，339.2185，385.2586	Isomer of cosmosporaside A
25	7.38	403.2483	$C_{24}H_{36}O_5$	301.2170, 311.2014，333.2448，343.2275，361.2401	(3alpha,5beta)-3-hydroxy-6,7-dioxocholan-24-oic acid
26	8.38	463.2903	$C_{23}H_{44}O_9$	255.2326，269.2486，297.2440	(2R)-3-(beta-D-Galactopyranosyloxy)-2-hydroxypropyl myristate
27	8.38	417.2852	$C_{22}H_{42}O_7$	59.0131，255.2331，269.2509,297.2439，309.2440	Polygonimitin B
28	8.51	473.2905	$C_{23}H_{22}O_{11}$	160.0174,175.0405,297.2452,473.2906	Aloe-emodin-8-O-(6'-O-acetyl)-glucoside
29	1.45	353.0706	$C_{16}H_{18}O_9$	111.0081,87.0090,173.0090,353.0722	Caffeoylquinic acid
30	1.47	531.1565	$C_{26}H_{26}O_{12}$	96.9606,194.9278,485.1667	Pyscion-8-O-(6-O-malonyl)-hexose
31	4.48	243.1965	$C_{14}H_{12}O_4$	163.1095,201.1201,225.1101	Oxyresveratrol
32	6.18	393.1946	$C_{19}H_{22}O_9$	79.9566,80.9649,207.0643,263.0944,313.1055	Hydroxymusizin-hexose
33	6.26	331.0651	$C_{13}H_{16}O_{10}$	140.1161,139.1120,170.1735,211.1337,213.1397,229.1455	Galloyl-hexose
34	6.38	315,0763	$C_{13}H_{16}O_9$	57.0343,81.0342,125.0234,179.0343,205.0862,214.0619	Protocatechuic acid hexose

35	6.45	489.3434	$C_{26}H_{50}O_8$	429.3224，489.3432	Methyl-3-(beta-D-glucopyranosyloxy)-2,16-dimethylheta decanoate
36	6.48	476.2748	-	78.9588,152.9954,717.0059,207.0650,402.2399,476.2764	-
37	6.48	313.1056	$C_{18}H_{18}O_5$	163.0763,179.0701,192.0424,207.0644	3-(3-Hydroxy-4-methoxybenzyl)-7-methoxy-2,3-dihydro-4H-chromen-4-one
38	6.58	431.3374	$C_{24}H_{48}O_6$	243.1596,413.3272,431.3358	Steary-D-glucopyranoside
39	6.64	327.1808	$C_{17}H_{28}O_6$	221.1910,239.2012,265.1797	Spiculisporic acid
40	6.76	331.1756	$C_{16}H_{28}O_7$	87.0084,197.1906,225.1853,251.1645	2-Isopropyl-5-methyllcyclohexyl beta-L-glucopyranosiduronic acid
41	7.02	327.1439	$C_{19}H_{20}O_5$	149.0240,178.0636,206.0576,327.1233	3-（3,4-Di methoxybenzyl）-7-methoxy-2,3-dihydro-4H-chromen-4-one
42	7.32	297.2434	$C_{18}H_{34}O_3$	183.1387,197.1519,279.2339	Ricinelaidic acid
43	7.33	614.330	-	152.9949,255.2297,452.2733,494.2832,614.3234	-
44	7.34	269.2121	$C_{15}H_{10}O_5$	89.0200,181.1648,225.2248,269.2303,	Emodin
45	7.39	343.1761	$C_{17}H_{28}O_7$	139.0036,179.1799,195.1758,239.1649,266.15181	3-hydroxy-13-tetradecene-1,3,4-tricarboxylic acid
46	7.66	265.1805	$C_{16}H_{26}O_3$	80.0264,124.0159,165.1647,221.1904,265.1813	(2-Dodecen-1-yl)succinic anhydride
47	7.96	441.0947	$C_{22}H_{18}O_{10}$	71.0132,113.0248,279.2505,321.2423,333.2438,380.2796	Epicatechin-O-gallate
48	8.29	269.2116	$C_{16}H_{30}O_3$	223.2069,225.2226,269.2116,	Emodin
49	8.32	257.2120	$C_{15}H_{30}O_3$	209.1919,211.2069,257.2119	15-Hydroxypentadecanoic acid/ Saccharopine
50	8.34	465.3043	$C_{21}H_{22}O_{12}$	59.0139,163.0609,255.2323,375.2900	Pentahydroxystilbene-O-hexose<br>-
51	8.45	473.2095	$C_{28}H_{42}O_6$	175.0397,297.2435,441.2677,473.2913	Aloe-emodin-8-O-(6'-O-acetyl)-glucoside<br>-

52	8.66	391.2488	-	80.9171,152.9954,255.2326,318.2780,391.2544	-
53	8.77	271.2271	$C_{16}H_{32}O_3$	223.2065,225.2210,271.2269	Juniperic acid
54	1.34	549.1665	$C_{26}H_{30}O_{13}$	89.0240,179.0556,221.0666,323.0981	Liquiritigenin-hexose-xyl/ara
55	1.38	784.2700	-	160.0609,280.1026,442.1567,665.2136	-
56	1.39	280.1036	-	68.0503,118.0501,160.0607	-
57	1.40	827.2616	$C_{40}H_{44}O_{19}$	179.0558,341.1084,545.1719	Polygonumosides C
58	1.43	179.0564	-	59.0135,71.0134	-
59	1.43	665.2137	$C_{24}H_{42}O_{21}$	161.0454,179.0556,221.0667,341,1085,665.2147	Isomer of alpha-maltotetraose
60	1.43	439.1207	$C_{20}H_{24}O_{11}$	78.9588,79.9571,96.9657,179.0554	-
61	1.44	549.1665	$C_{19}H_{34}O_{18}$	161.0457,179.0556,221.0672,341.1089,503,1607	-
62	1.46	503.1610	$C_{18}H_{32}O_{16}$	89.0240,179.0559,161.0454,221.0668,503.1640	Isomer of maltotriose
63	1.46	647.2043	$C_{24}H_{40}O_{20}$	89.0241,179.0559,323.0987,485.1525,647.2033	-
64	1.48	485.1508	$C_{18}H_{30}O_{15}$	89.0240,113.0243,161.0454,179.0562,485.1535	-
65	1.48	531.1565	$C_{19}H_{32}O_{17}$	89.0242,161,0454,179.0563,323.0982,485.1505	-
66	1.50	539.1385	$C_{24}H_{28}O_{14}$	89.0242,161.0451,179.0556,221.0668	Centaurein monohydrate
67	2.40	345.2643	$C_{14}H_{18}O_{10}$	59.0164,225.1938,255.2317,299.2572,299.2831	Polygoacetophenoside
68	5.79	312.1222	$C_{18}H_{19}NO_4$	135.0451,148.0522,190.0505,178.0503,178.0503	-
69	6.02	365.0682	-	119.0515,165.0557,245.0114,365.0686	-
70	6.26	331.2468	$C_{14}H_{20}O_9$	139.1120,140.1161,171.1035,211.1337,213.1397,	Gallic acid hexose
71	6.89	339.2167	$C_{19}H_{32}O_5$	185.1182,209.1171,245.1916,289.1811,307.1916	Isomer of 3alpha-hydroxy-3,5-dihydromonacolin L acid
72	7.41	293.2112	$C_{18}H_{30}O_3$	113.0968,139.1128,195.1385,249.2228, 293.2113	Isomer of 12-oxo-octadecynoic acid
73	7.88	279.2324	$C_{18}H_{32}O_2$	261.2207.279.2319	Linoleic acid
74	8.29	269.2116	$C_{16}H_{30}O_3$	225.2199.269.2110	Isomer of (7E)-16-hydroxy-7-hexadecenoic acid
75	8.63	389.1994	$C_{20}H_{22}O_8$	78.9201,80.9560,278.2489,291.1914,309.2073	Resveratrol-hexose
76	9.08	555.2835	-	152.9948,255.2343,409.2399,555.2901	-

77	9.15	555.2838	$C_{27}H_{24}O_{13}$	225.0060,255.2310,555.1492	Isomer of Polygonumosidesl A
78	0.22	179.0519	$C_9H_8O_4$	59.0141,71.0158,71.0276	Caffeic acid
79	1.08	219.1754	-	61.9877,79.9577,99.9261,116.9268,219.1761	-
80	1.10	833.5164	$C_{42}H_{42}O_{18}$	279.2307,391.2253,833.5575	Di-emodin-Di-hexose
81	1.44	693.2092	$C_{35}H_{34}O_{15}$	179.0565,323.0987,485.1516,647.2037	Polyginflavanol A

Table S2 The represent mark for each sample of R-PCH and P-PCHs.

**No.**	**Rt (min)**	***m/z* (Neg)**	**Formula**	**p-value**	**Fold Change**	**Log (Fold Change)**
1[a]	7.39	403.2483	$C_{24}H_{36}O_5$	0.0002	0.0017	–2.7617
2[b]	6.81	373.1264	$C_{20}H_{22}O_7$	0.0058	84.2403	1.9742
3[c]	6.59	431.3374	$C_{24}H_{48}O_6$	0.00143	0.003544	–2.4505
4[d]	1.43	622.2197	$C_{30}H_{33}N_5O_{10}$	0.00136	38.19893	1.5820
5[e]	7.06	913.4755	$C_{47}H_{74}O_{18}$	0.00035	32.4771	1.5116
6[f]	7.96	767.4222	$C_{51}H_{60}O_6$	0.0000244	35.27765	1.5475
7[g]	8.43	425.2903	-	0.00519	47.58022	1.6774
8[h]	7.47	267.1603	$C_{15}H_{24}O_4$	0.00044	0.022763	–1.6428
9[i]	7.77	399.2517	$C_{25}H_{36}O_4$	0.0004	0.028315	–1.5480
10[j]	6.64	327.1808	$C_{18}H_{32}O_5$	0.0003	0.0044	–2.3535

a is behalf on the maker specific for R-PCH; b~i are behalf on the makers specific for P-PCH I~IX, respectively.

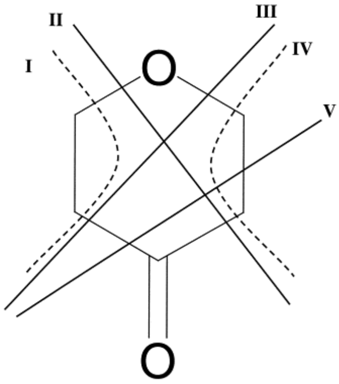

Figure S1 The proposed fragmental patterns of C ring for flavonoids.

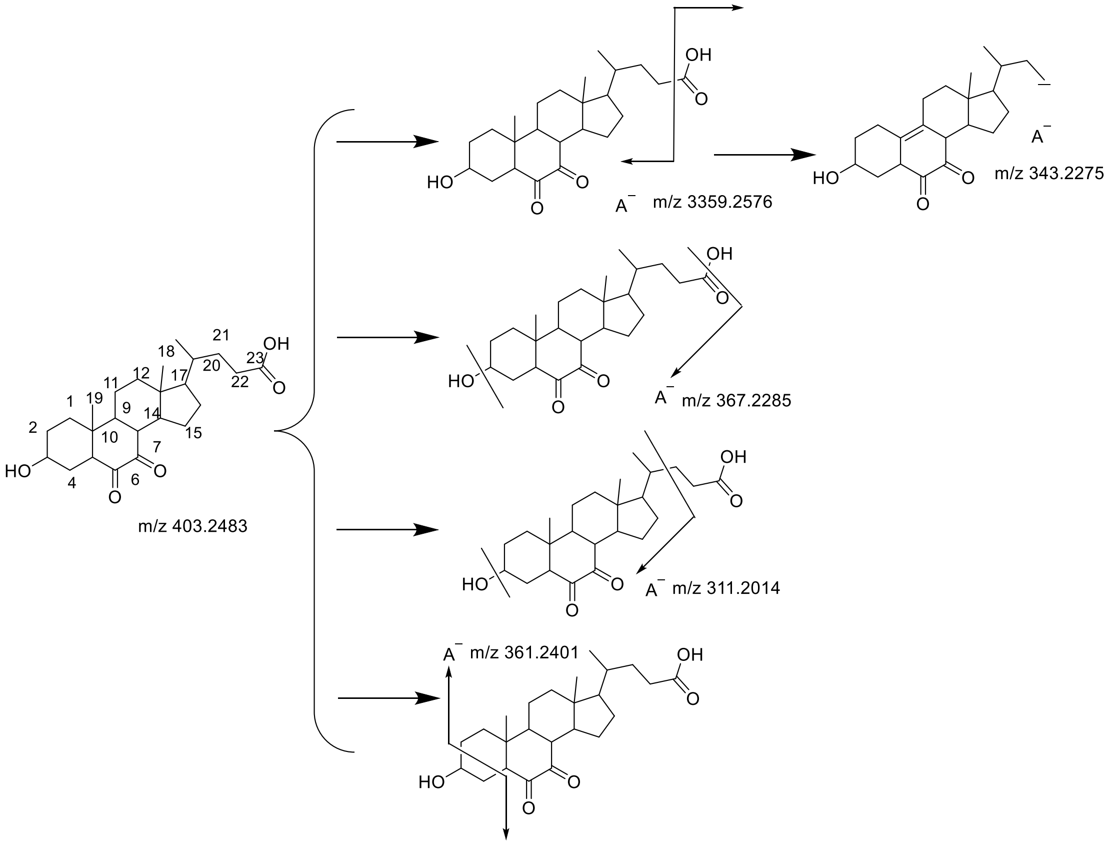

Figure S2 The plausible fragmentation pathways of compound **3.**

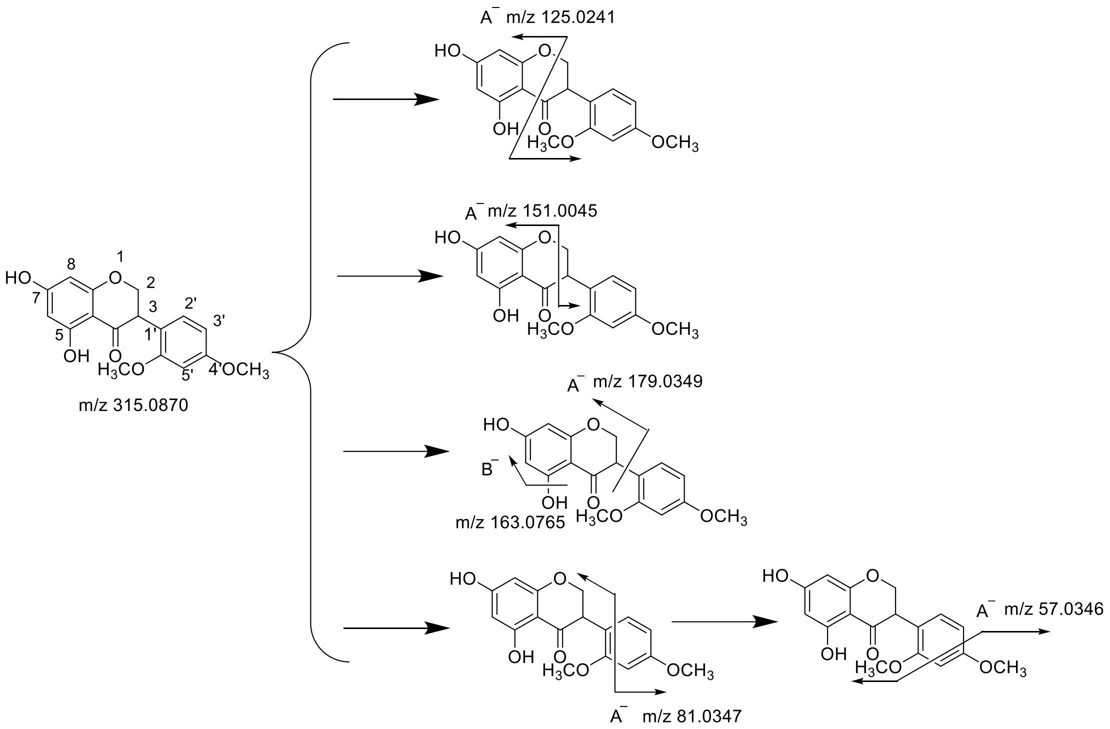

Figure S3 The plausible fragmentation pathways of Homoferreirin.

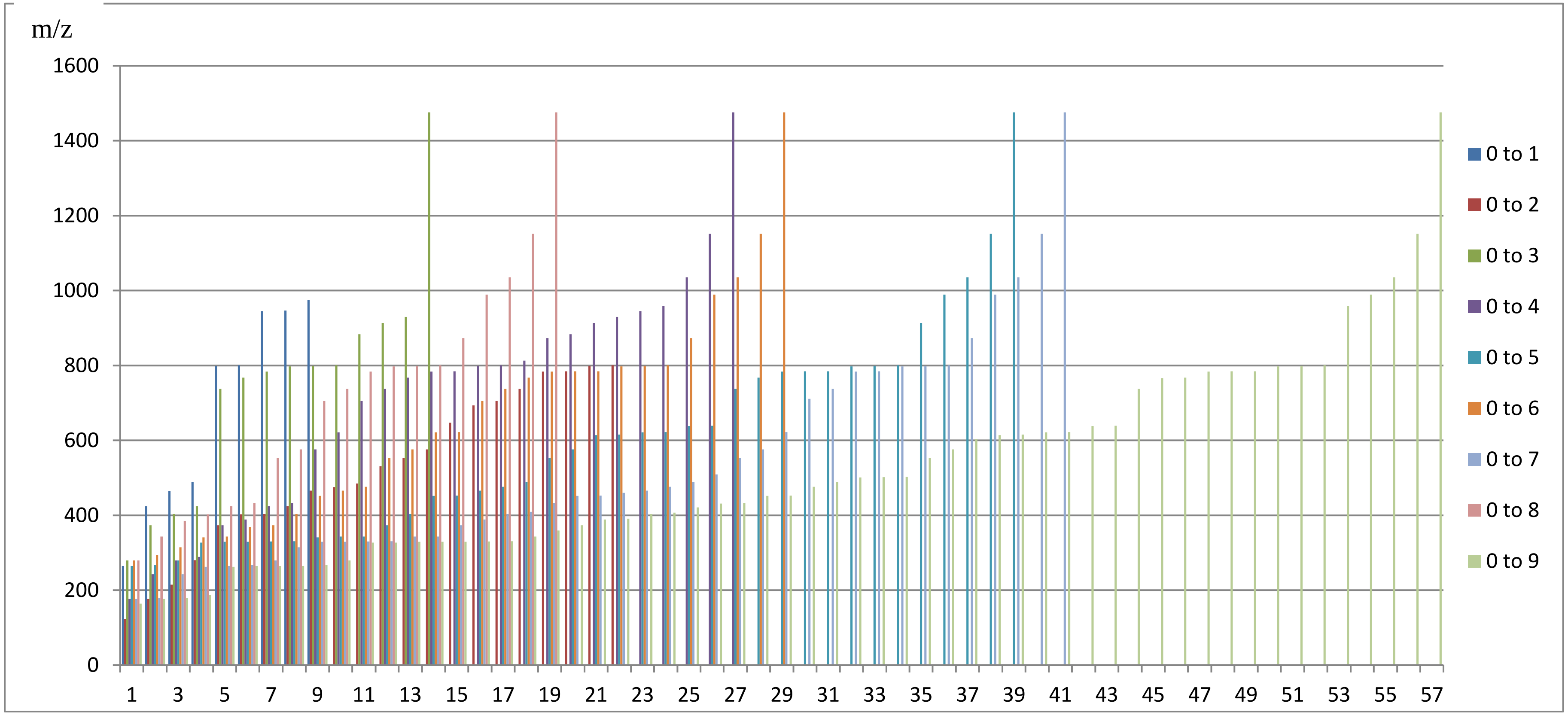

Figure S4 The chemical markers for discrimination of R-PCH and other P-PCHs

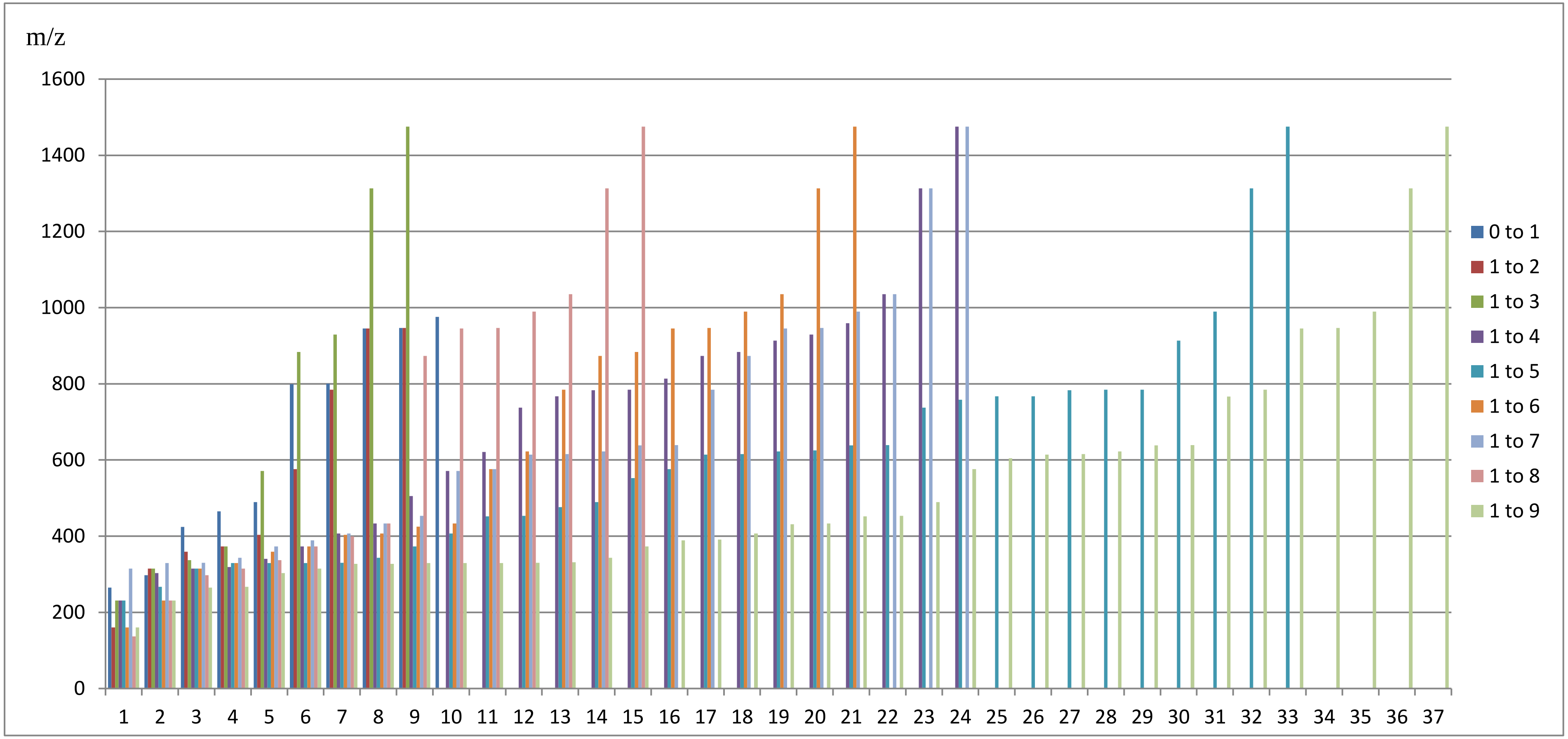

Figure S5 The chemical markers for discrimination of PCH-I and other P-PCHs (including R-PCH)

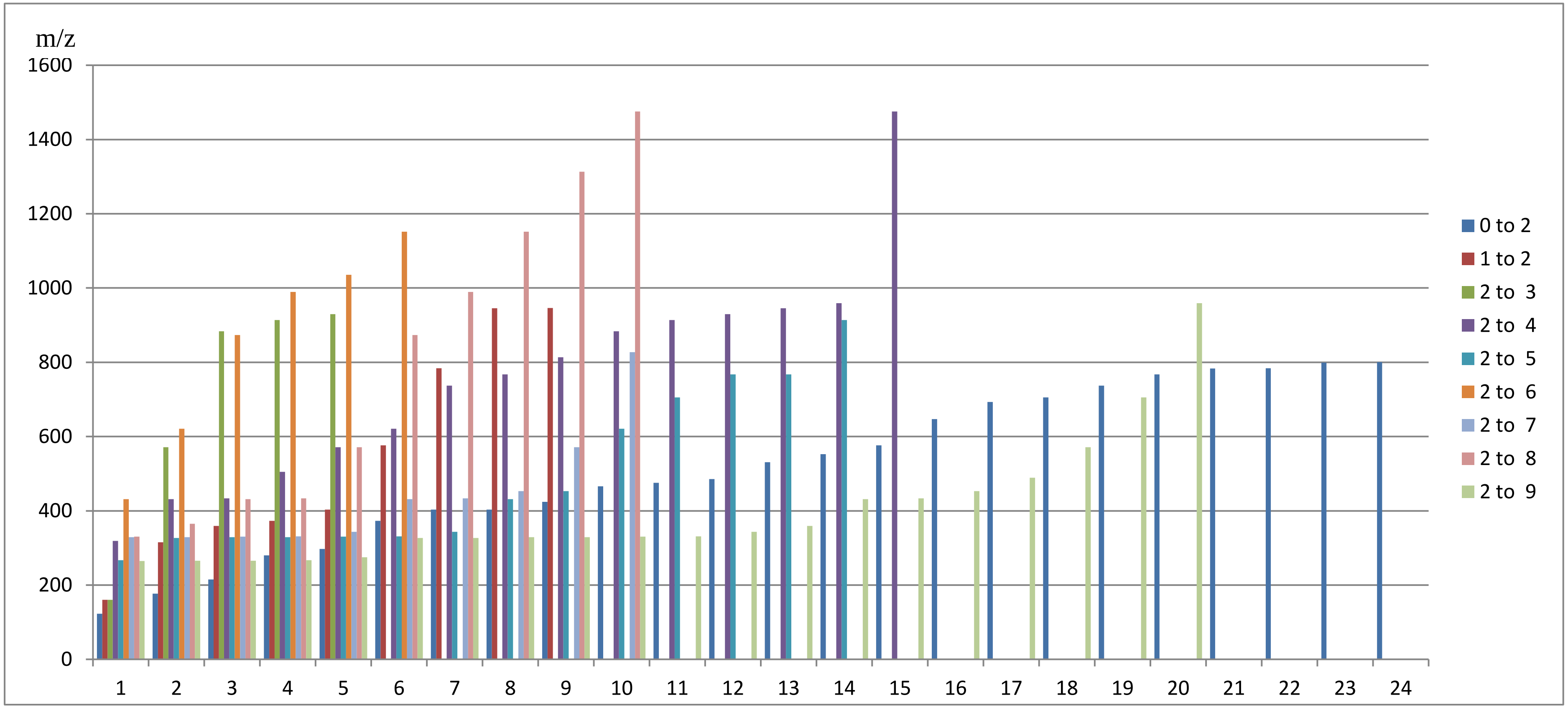

Figure S6 The chemical markers for discrimination of PCH-II and other P-PCHs (including R-PCH)

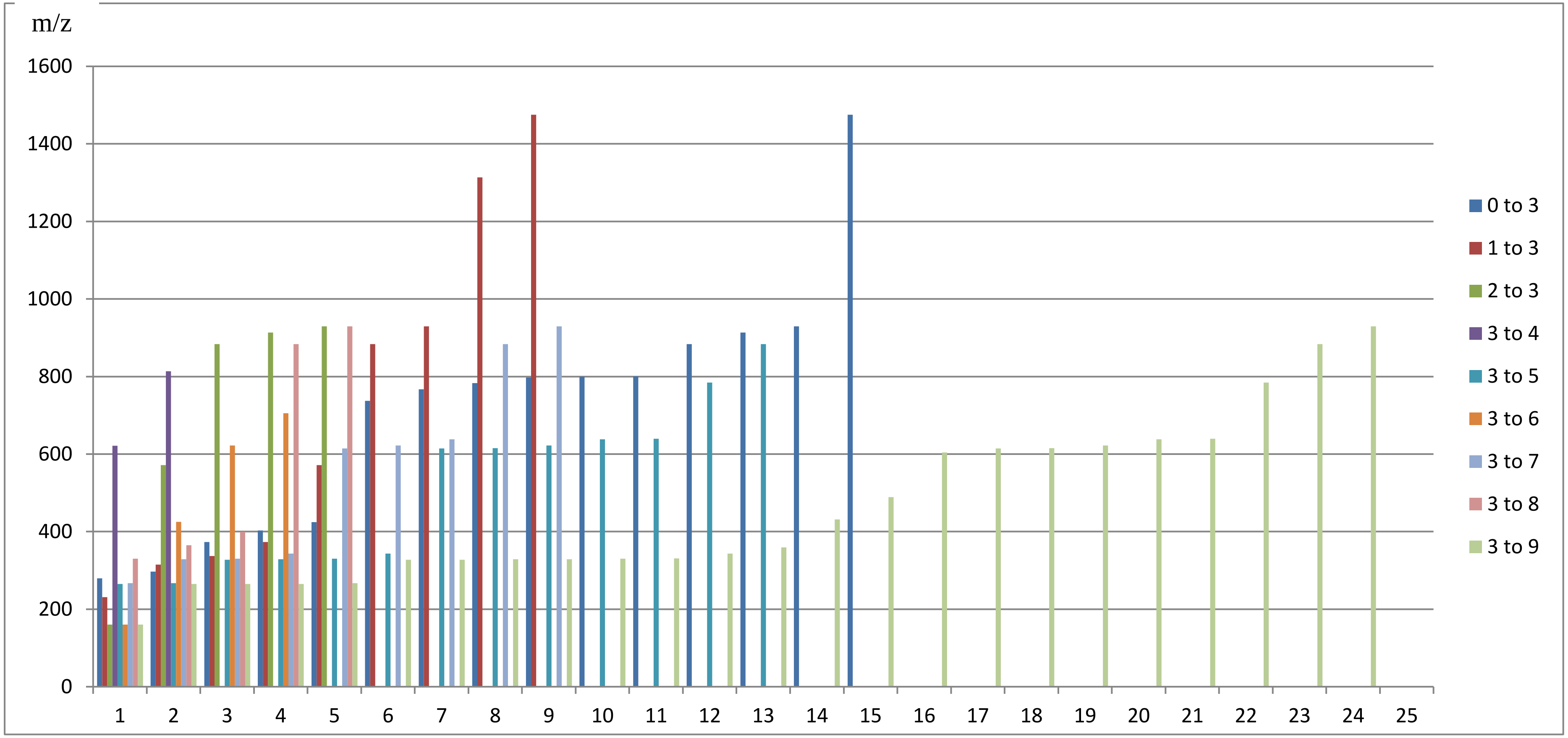

Figure S7 The chemical markers for discrimination of PCH-III and other P-PCHs (including R-PCH)

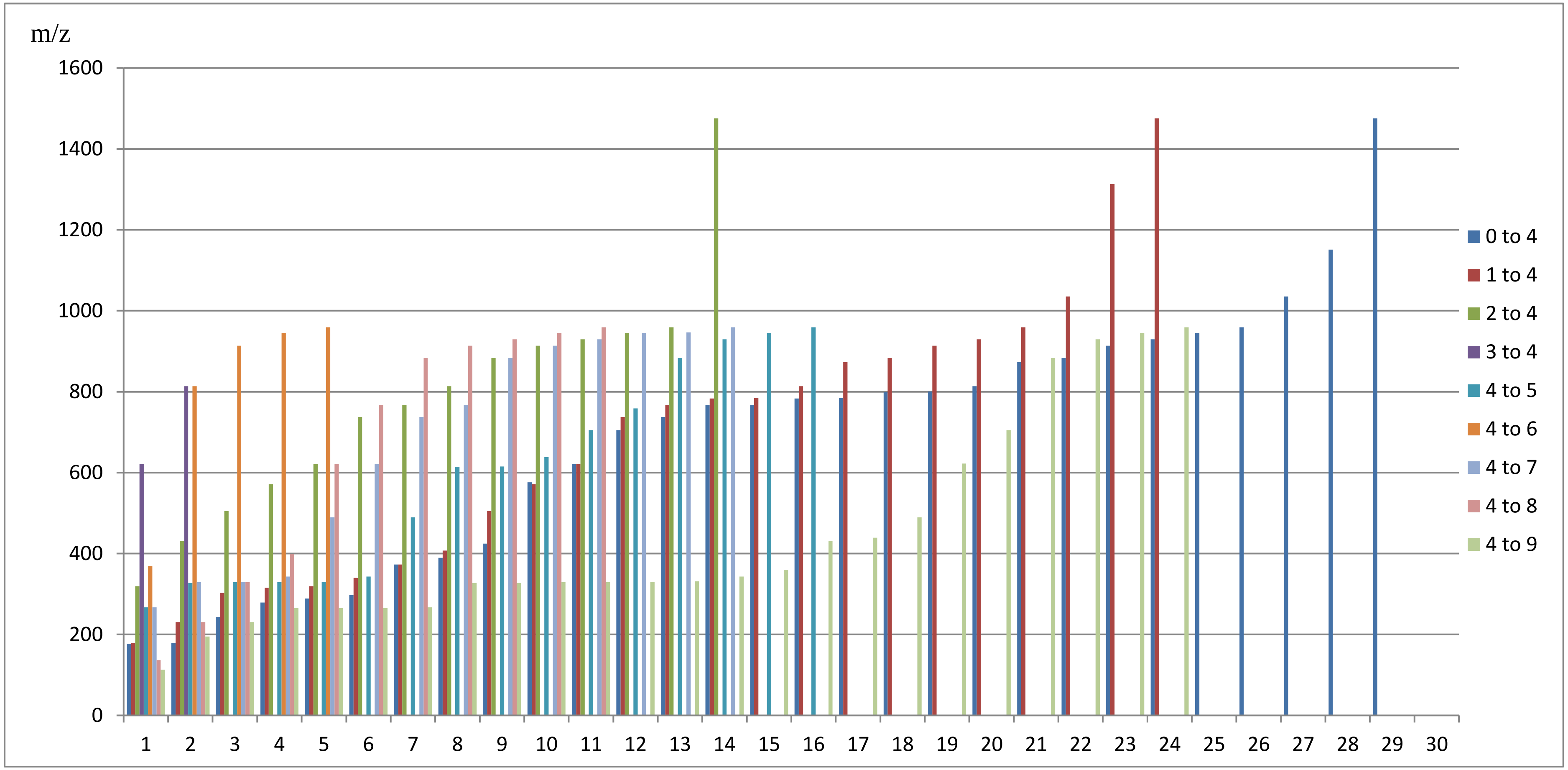

Figure S8 The chemical markers for discrimination of PCH-IV and other P-PCHs (including R-PCH)

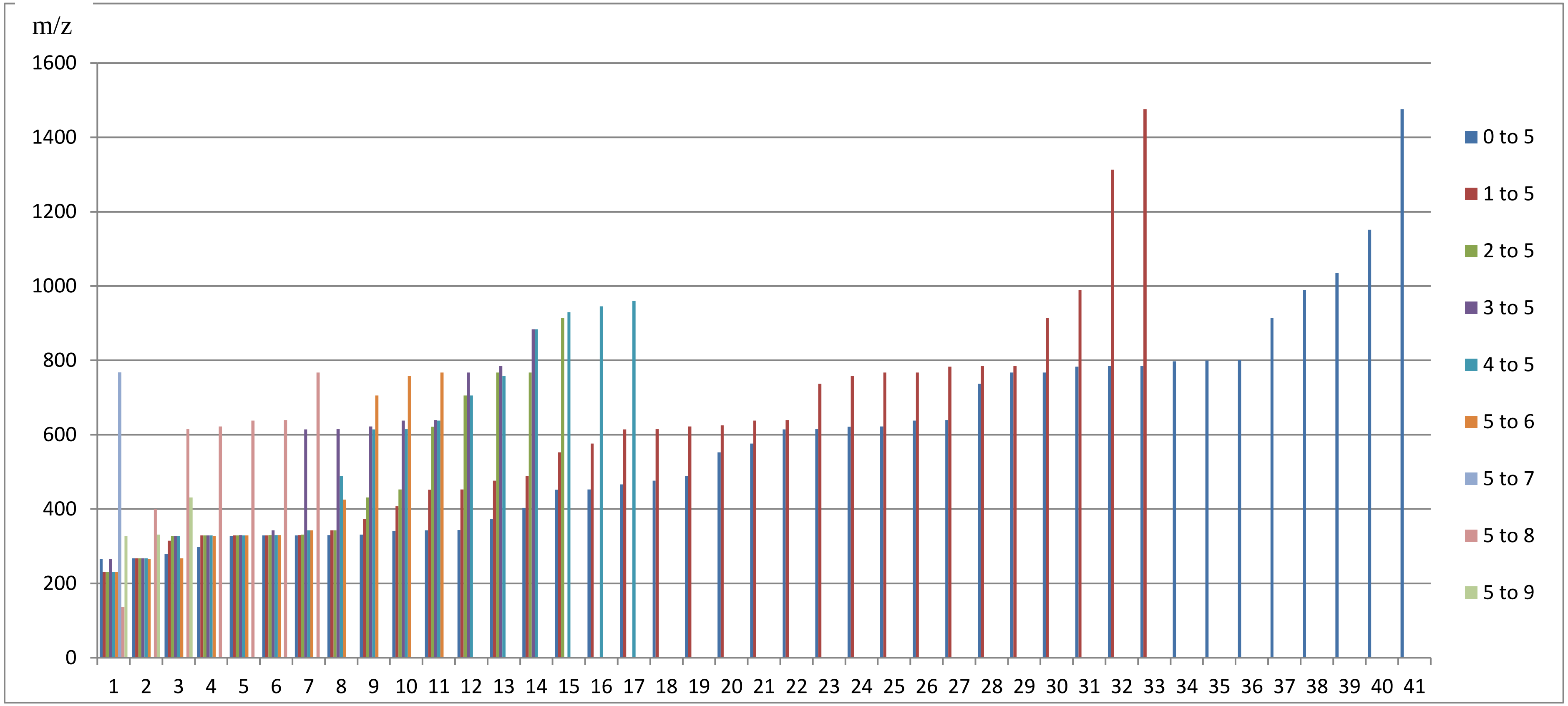

Figure S9 The chemical markers for discrimination of PCH-V and other P-PCHs (including R-PCH)

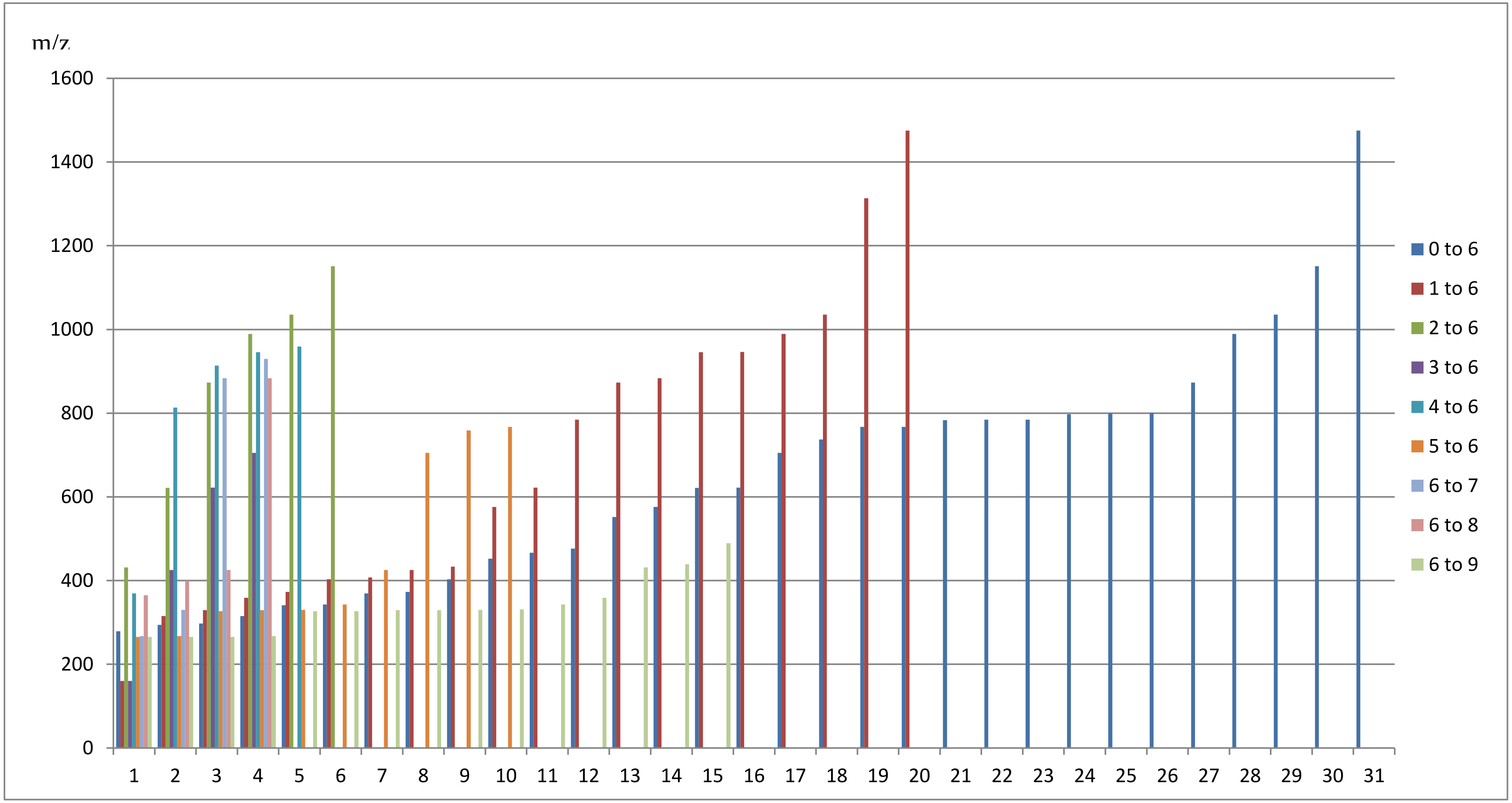

Figure S10 The chemical markers for discrimination of PCH-VI and other P-PCHs (including R-PCH)

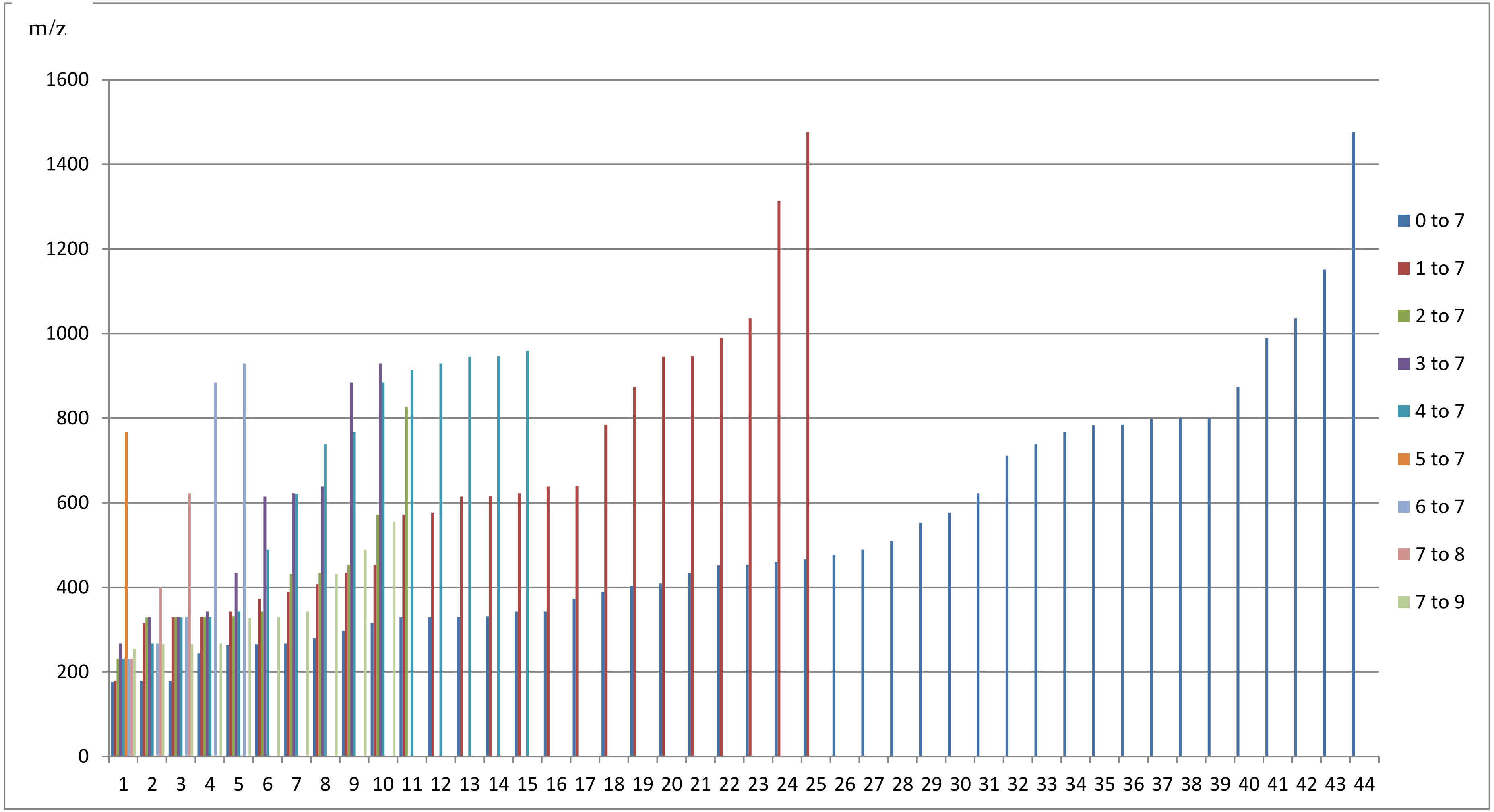

Figure S11 The chemical markers for discrimination of PCH-VII and other P-PCHs (including R-PCH)

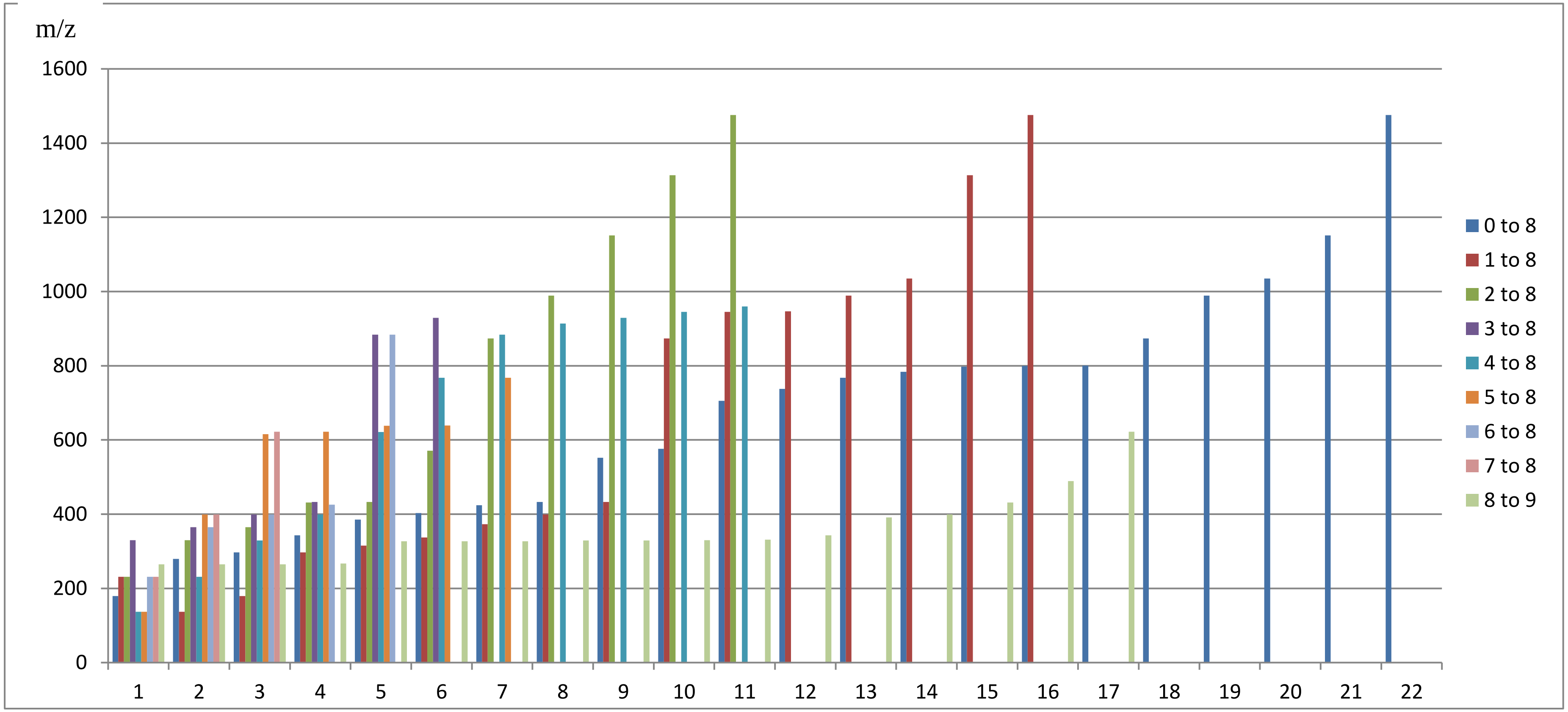

Figure S12 The chemical markers for discrimination of PCH-VIII and other P-PCHs (including R-PCH)

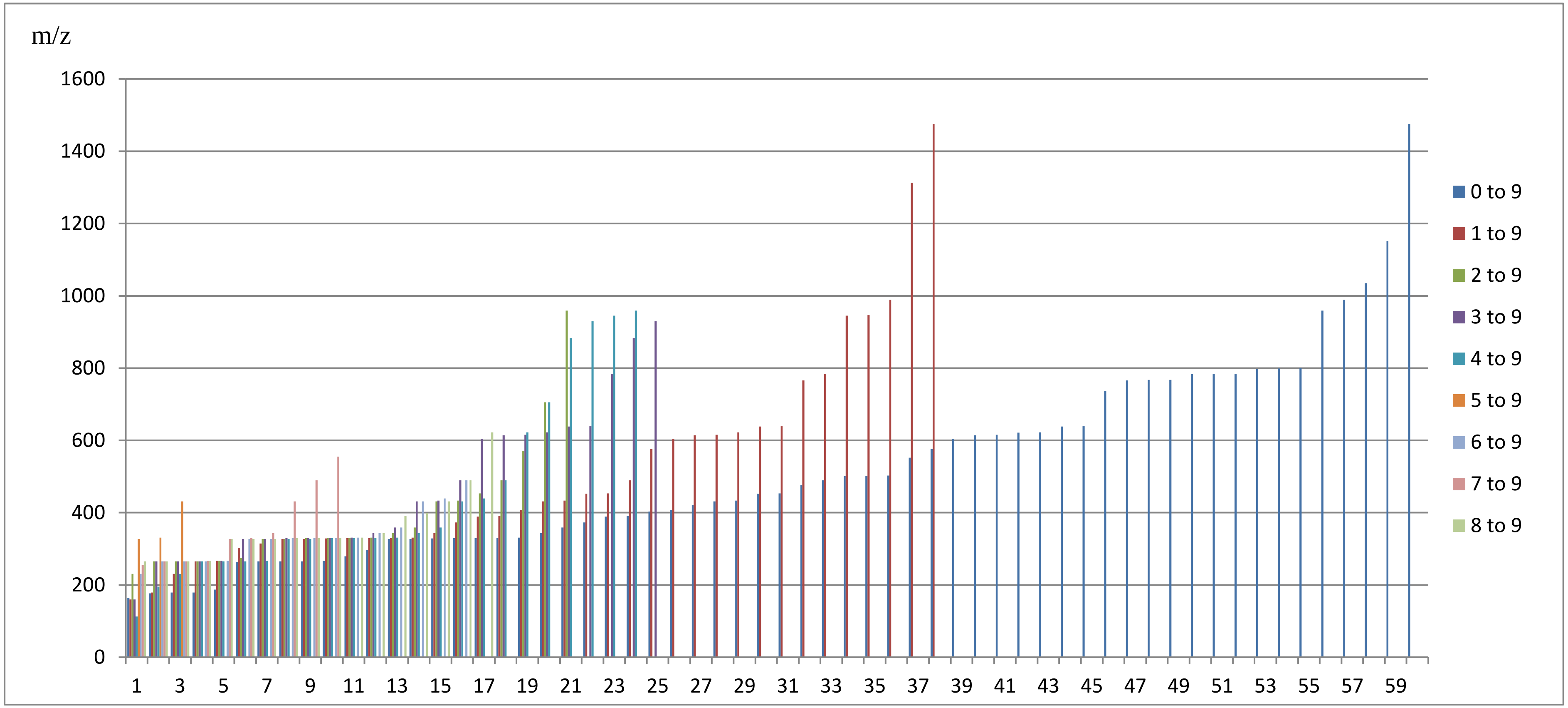

Figure S13 The chemical markers for discrimination of PCH-IX and other P-PCHs (including R-PCH)

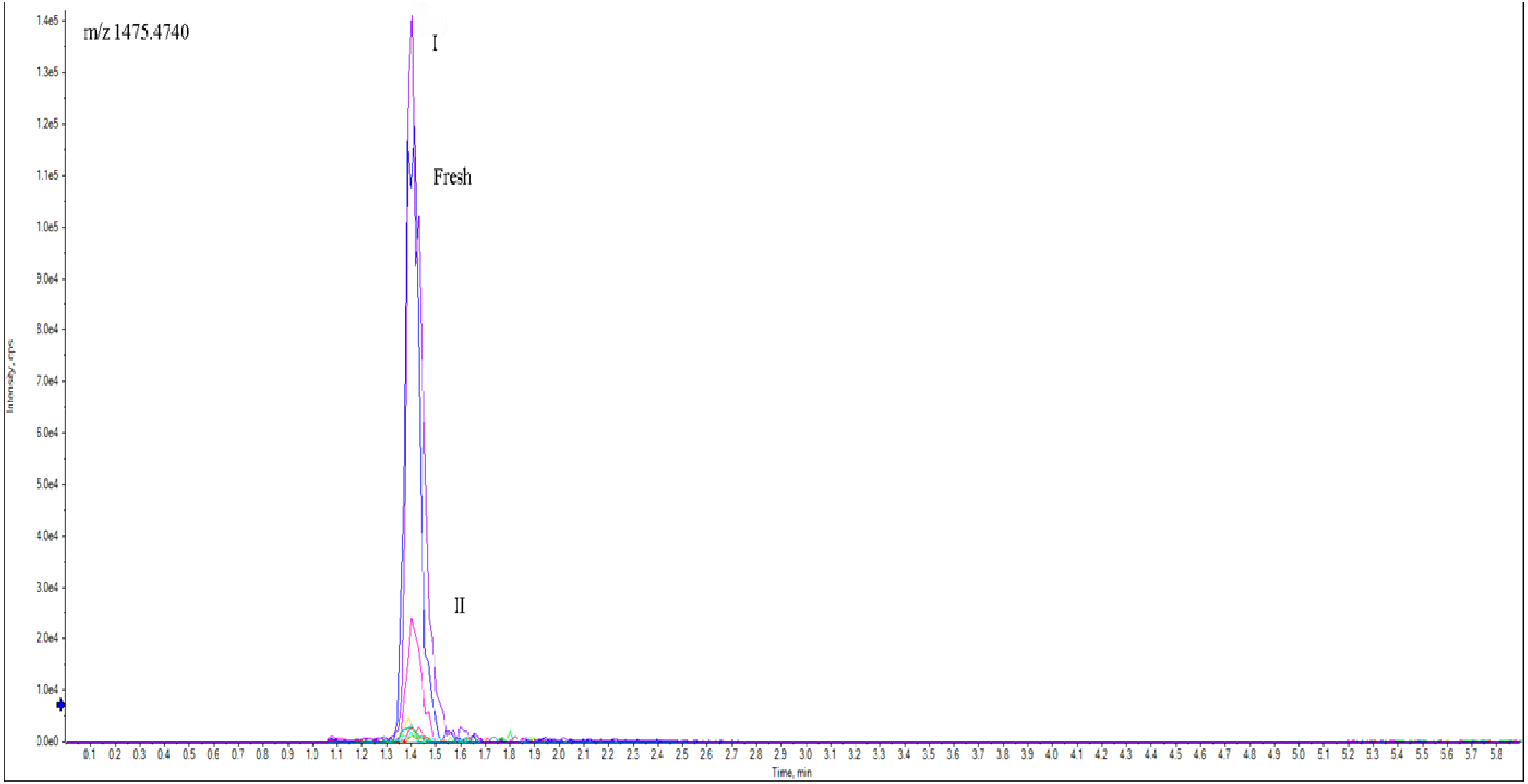

Figure S14 The extract ion chromatogram (XIC) of the marker 1475.4740 in thereof different P-PCHs and R-PCH, to clarify the dynamic changes in the process of steaming and basking.

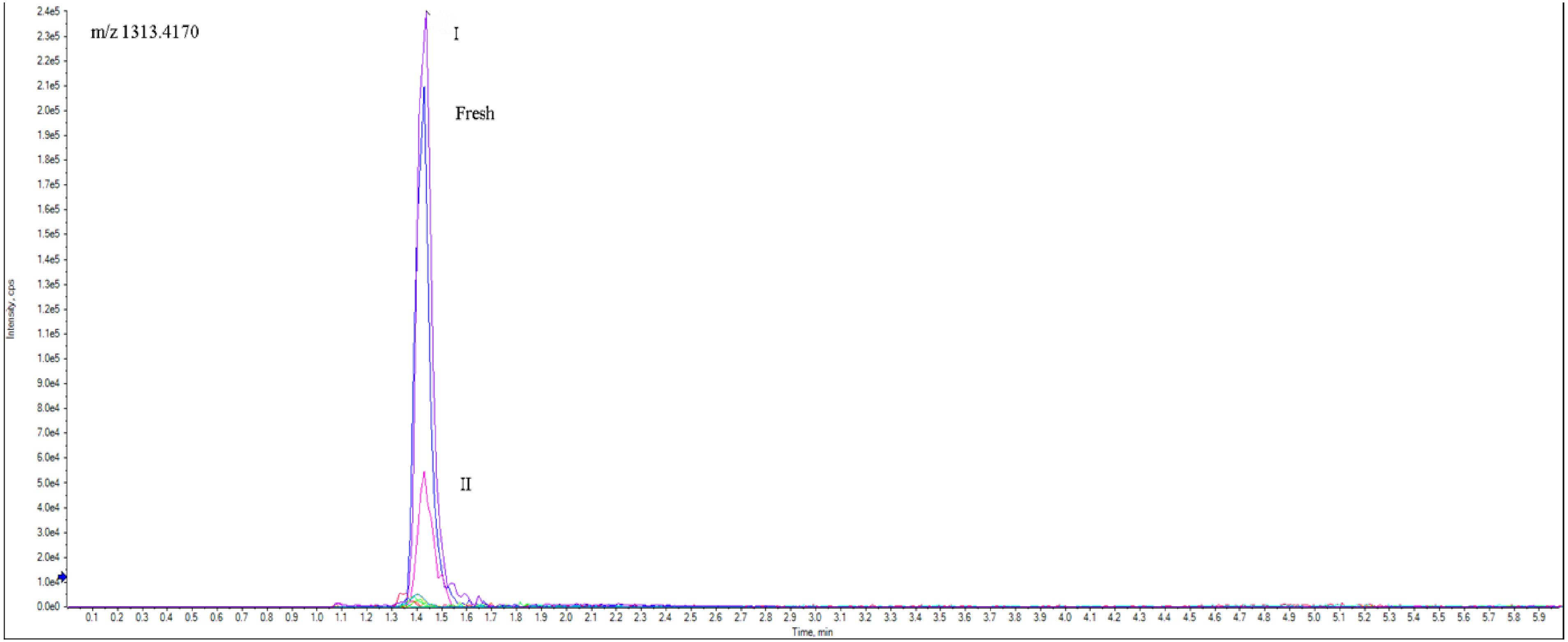

Figure S15 The extract ion chromatogram (XIC) of the marker 1313.4170 in thereof different P-PCHs and R-PCH, to clarify the dynamic changes in the process of steaming and basking.

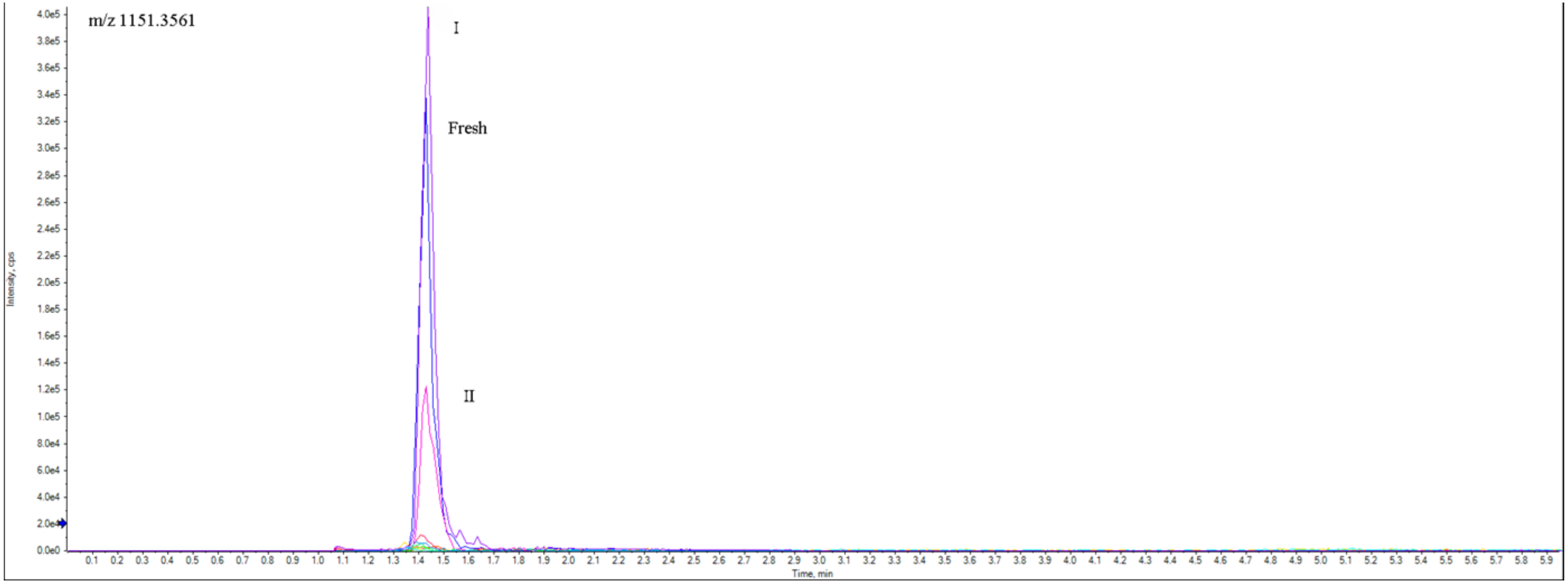

Figure S16 The extract ion chromatogram (XIC) of the marker 1151.3561 in thereof different P-PCHs and R-PCH, to clarify the dynamic changes in the process of steaming and basking.

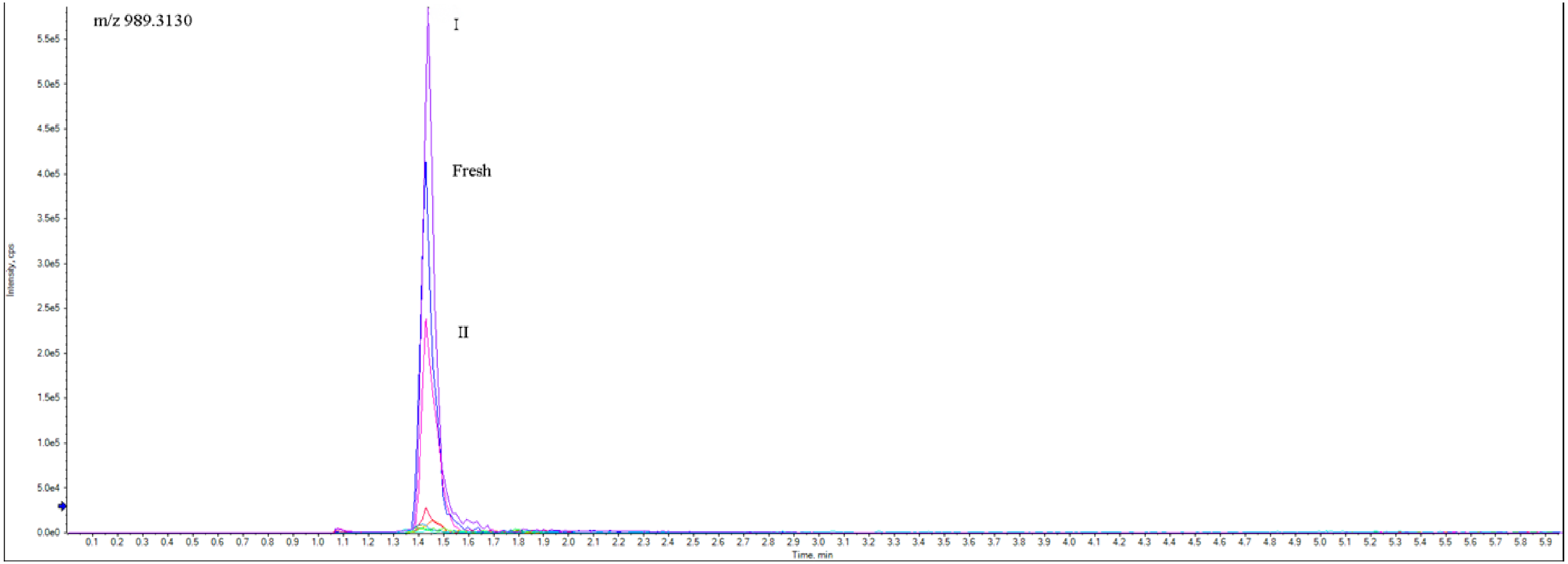

Figure S17 The extract ion chromatogram (XIC) of the marker 989.3130 in thereof different P-PCHs and R-PCH, to clarify the dynamic changes in the process of steaming and basking.

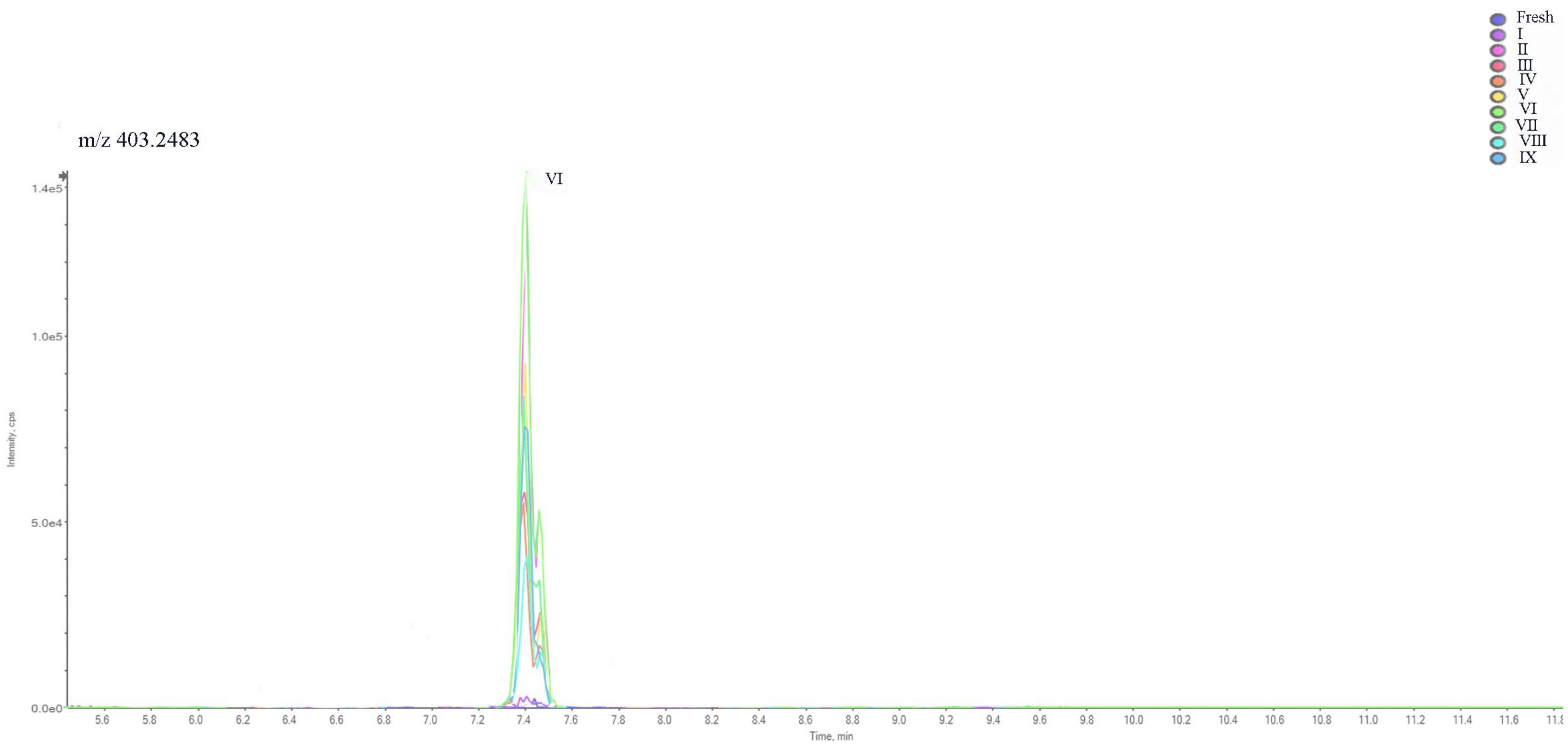

Figure S18 The extract ion chromatogram (XIC) of the marker 403.2483 in thereof different P-PCHs and R-PCH, to clarify the dynamic changes in the process of steaming and basking.

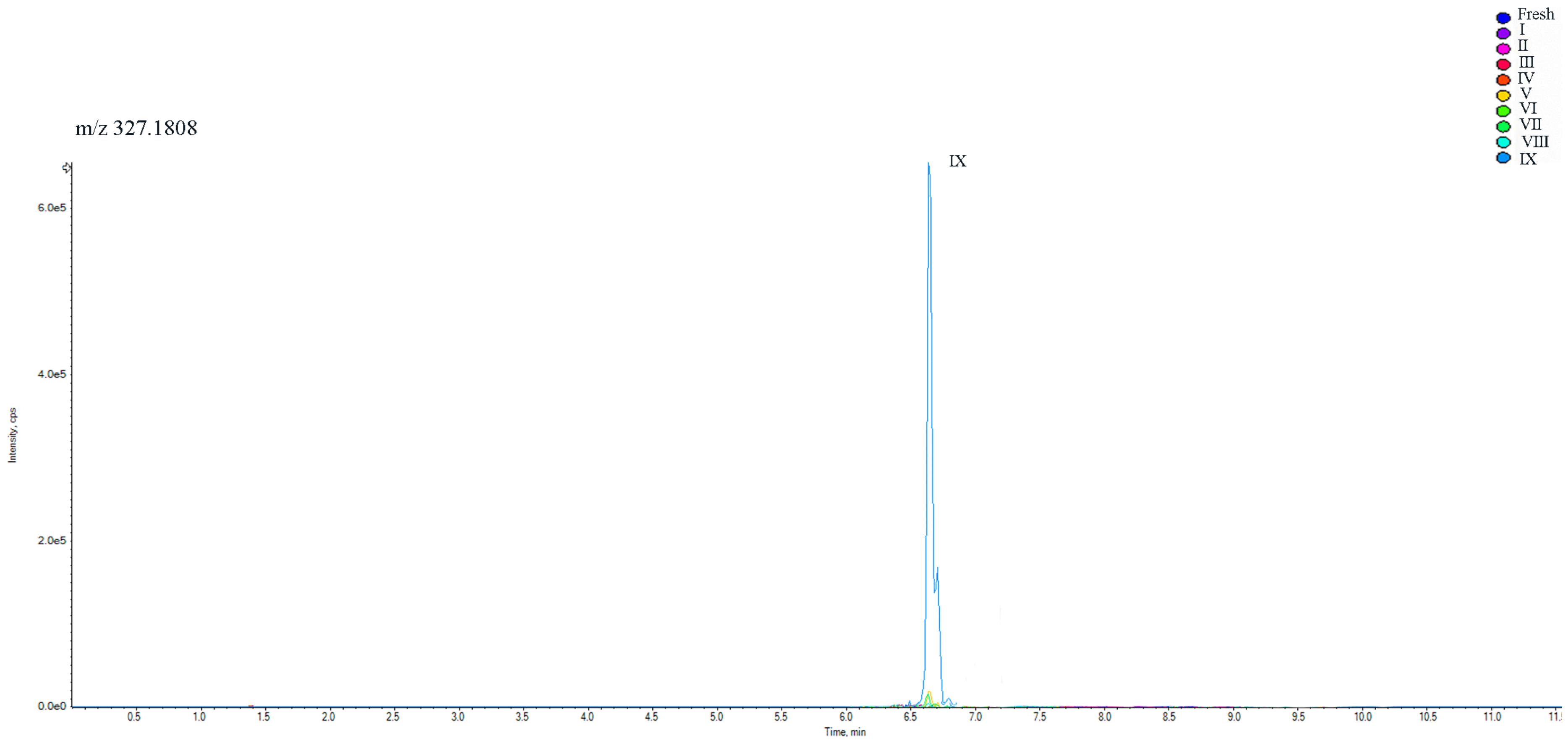

Figure S19 The extract ion chromatogram (XIC) of the marker 327.1808 in thereof different P-PCHs and R-PCH, to clarify the dynamic changes in the process of steaming and basking.

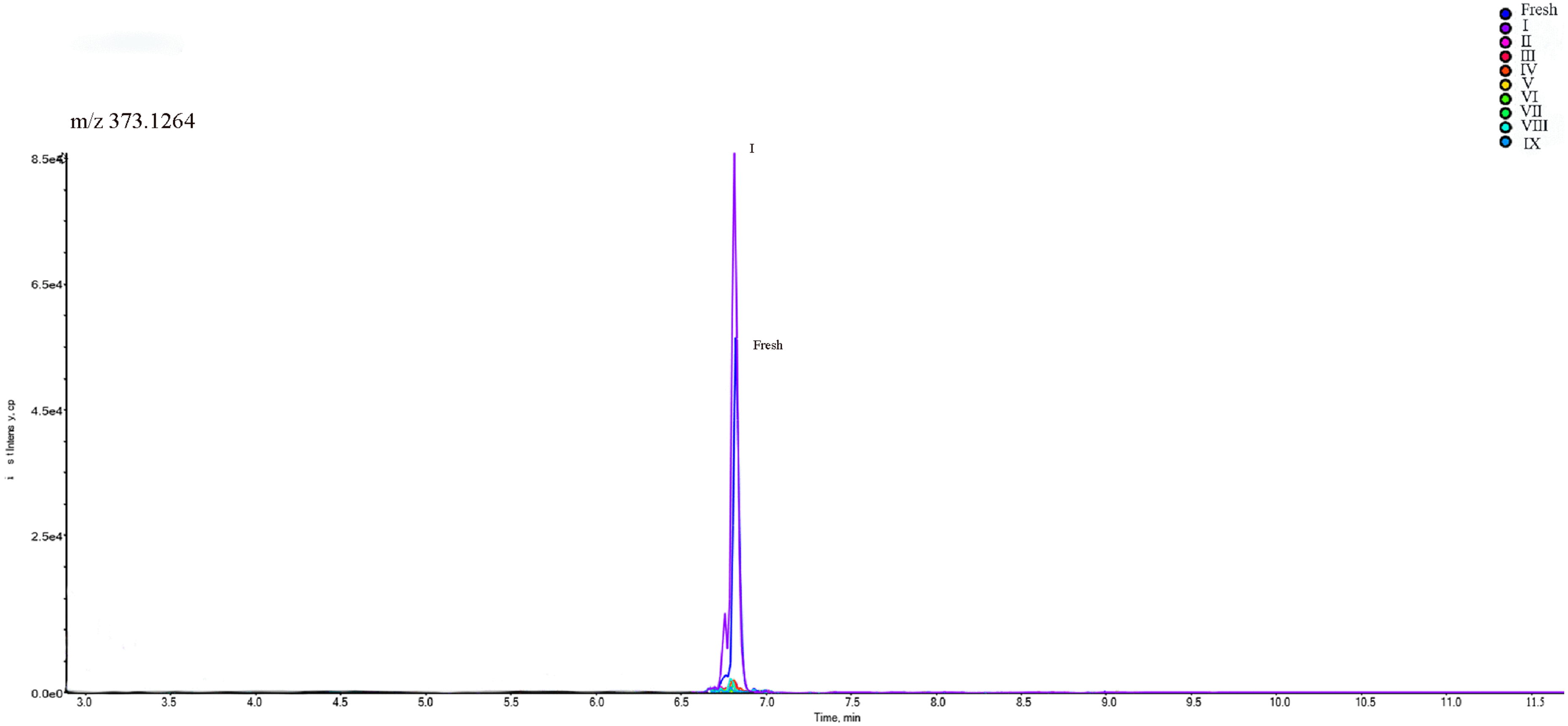

Figure S20 The extract ion chromatogram (XIC) of the marker 373.1264 in thereof different P-PCHs and R-PCH, to clarify the dynamic changes in the process of steaming and basking.

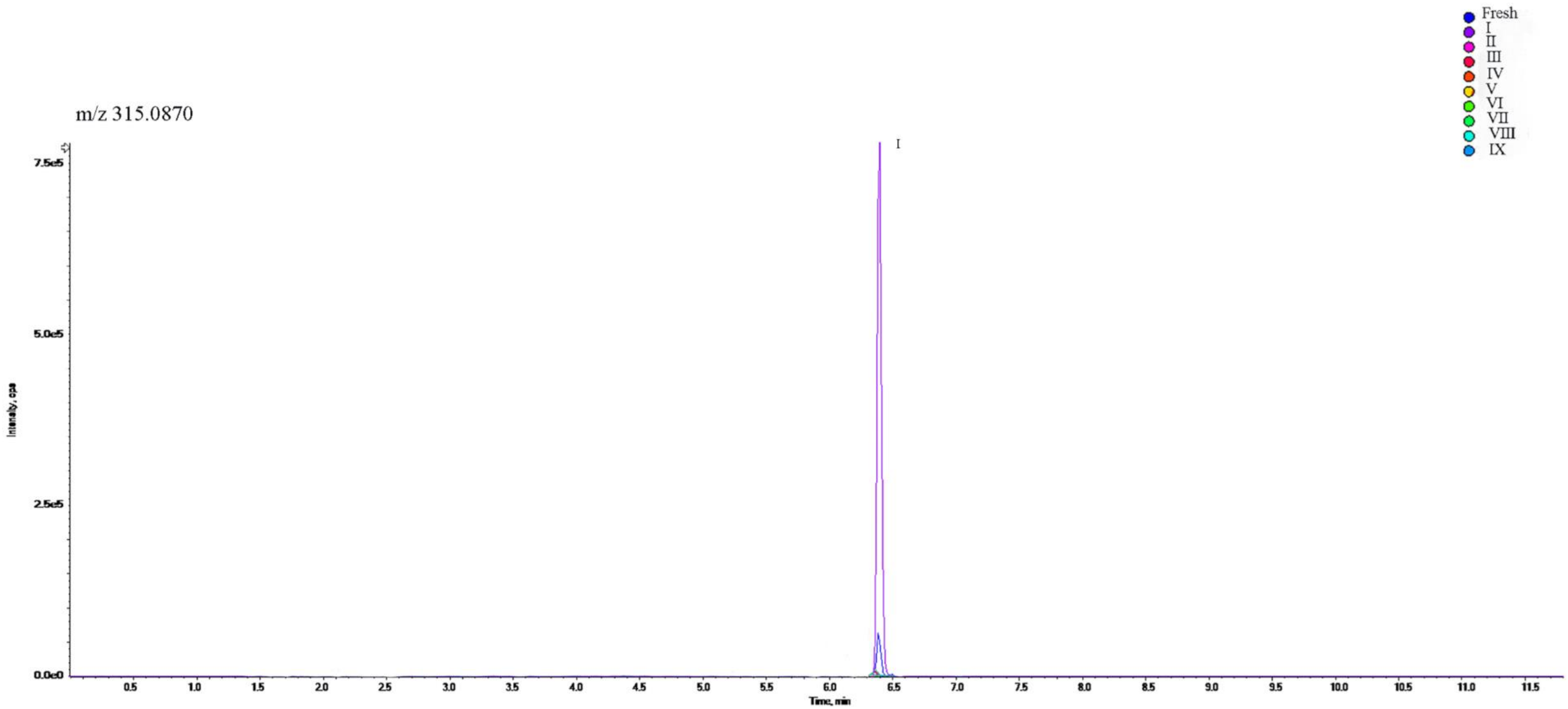

Figure S21 The extract ion chromatogram (XIC) of the marker 315.0870 in thereof different P-PCHs and R-PCH, to clarify the dynamic changes in the process of steaming and baskin
